# Regulating electronic states of nitride/hydroxide to accelerate kinetics for oxygen evolution at large current density

**DOI:** 10.1038/s41467-023-37091-x

**Published:** 2023-04-04

**Authors:** Panlong Zhai, Chen Wang, Yuanyuan Zhao, Yanxue Zhang, Junfeng Gao, Licheng Sun, Jungang Hou

**Affiliations:** 1 State Key Laboratory of Fine Chemical, Frontiers Science Center for Smart Materials Oriented Chemical Engineering, School of Chemical Engineering, Dalian University of Technology, Dalian, 116024 P. R. China; 2 State Key Laboratory of Structural Analysis, Optimization and CAE Software for Industrial Equipment, Dalian University of Technology, Dalian, 116024 P. R. China; 3 Center of Artificial Photosynthesis for Solar Fuels and Department of Chemistry, School of Science, Westlake University, Hangzhou, 310024 P. R. China; 4 Department of Chemistry, School of Engineering Science in Chemical, Biotechnology and Health KTH Royal Institute of Technology, Stockholm, 10044 Sweden

**Keywords:** Electrocatalysis, Electrocatalysis, Electrocatalysis

## Abstract

Rational design efficient transition metal-based electrocatalysts for oxygen evolution reaction (OER) is critical for water splitting. However, industrial water-alkali electrolysis requires large current densities at low overpotentials, always limited by intrinsic activity. Herein, we report hierarchical bimetal nitride/hydroxide (NiMoN/NiFe LDH) array as model catalyst, regulating the electronic states and tracking the relationship of structure-activity. As-activated NiMoN/NiFe LDH exhibits the industrially required current density of 1000 mA cm^−2^ at overpotential of 266 mV with 250 h stability for OER. Especially, in-situ electrochemical spectroscopic reveals that heterointerface facilitates dynamic structure evolution to optimize electronic structure. Operando electrochemical impedance spectroscopy implies accelerated OER kinetics and intermediate evolution due to fast charge transport. The OER mechanism is revealed by the combination of theoretical and experimental studies, indicating as-activated NiMoN/NiFe LDH follows lattice oxygen oxidation mechanism with accelerated kinetics. This work paves an avenue to develop efficient catalysts for industrial water electrolysis via tuning electronic states.

## Abstract (web summary)

Rational design of efficient electrocatalysts for oxygen evolution reaction is critical for water-alkali electrolysis. Here, the authors fabricate a NiMoN/NiFe layered double hydroxide and show the accelerated oxygen evolution kinetics are due to the heterointerface.

## Introduction

With the intensification of the energy crisis and climate concerns, the development of renewable and clean resources has far-reaching significance for fossil fuel consumption and sustainable economic development. As a clean and reliable energy technology, hydrogen is regarded as an appropriate alternative to fossil fuel, while water electrolysis using intermittent electric energy represents a promising commercial technology for industrial hydrogen production^1,2^. Water splitting reaction consists of hydrogen evolution reaction (HER) at the cathode and oxygen evolution reaction (OER) at the anode. Compared with the HER, the OER has sluggish kinetics and a large reaction barrier, limiting the efficiency of electrocatalytic water splitting^3^. To address these points, the development of highly active oxygen evolution electrocatalysts has become a research hotspot. Pt and Ir/Ru oxide-based electrocatalysts are benchmark catalysts for water splitting; however, scarcity of reserves, high cost and inferior stability limit their large-scale application. To overcome these challenges, the exploration of transition metal-based electrocatalysts serviced in alkaline media, which simultaneously meets the requirements of intrinsic activity and stability, has attracted wide attention^4^.

To date, numerous strategies, such as composition tuning^5,6^, heteroatom doping^7,8^ and defect engineering^9,10^, have been reported for enhancing OER activity. Among various tactics, heterointerface engineering^11–16^ is one of the most considerable ways to overcome the limitation of catalytic activity and improve the intrinsic activity of electrocatalysts. The construction of heterostructure is conducive to the formation of the active phase and optimization of electronic structure, owing to the multi-component synergistic effect, in which creates catalytic sites and modulates intermediate adsorption^11,17,18^. For instance, Luo et al.^19^ designed a CoOOH/CoS_α_ hybrid catalysts, regulating the formation of high-valent metal species and enhancing the adsorption capacity of the intermediate. Du et al.^20^ synthesized NiO/NiFe LDH with excellent OER performance. In situ spectroscopies and theoretical calculations revealed the dynamic tridimensional adsorption of the intermediate at the heterointerface, which bypasses the scaling relationship and facilitates the reaction kinetics. Even though numerous efforts have been made, the structure-property relationships between heterostructure and catalytic performance need to be further analyzed, especially, the electronic configuration and reaction kinetics.

Although various advances have been obtained in performance and mechanistic understanding for OER, the progress in industrial application is still unsatisfactory^21–26^. The bottleneck hindering the progress of industrial water-alkali electrolysis is the rational design of catalysts towards practically-relevant current density (>1000 mA cm^−2^). It is desirable to synthesize transition metal-based and robust electrocatalysts, especially at large current densities. For instance, three-dimensional (3D) core-shell NiMoN@NiFeN catalysts present the current densities of 500 and 1000 mA cm^−2^ at the low voltages of 1.608 and 1.709 V for overall alkaline seawater splitting at 60 °C^21^. The EP Ni║EP NiFe LDH/Ni–cotton cell (electroplate noted as EP) show the cell voltages of 1.39, 1.63, and 1.81 V at the current densities of 10, 100, and 1000 mA cm^−2^ in 1 M KOH electrolyte^24^. Based on the previous literatures for industrial water electrolysis, the obvious conditions such as sufficient active sites, rapid diffusion of bubbles and good mechanically or chemically stability should be noticed^27,28^. In brief, we need rationally designed electrocatalysts to lay a solid foundation for industrial water electrolysis. Generally, OER process follows adsorbate evolution mechanism (AEM) where a series of sequential concerted proton-electron transfer (CPET) step occurs on metal sites via multiple oxygen intermediates. However, the thermodynamics constraint of the scaling relationship between the Gibbs free energy of *OOH and *OH cause the minimum theoretical overpotential of 0.37 V for the optimal catalysts^29^. More recently, the OER mechanism based on oxygen redox chemical was mentioned as lattice oxygen oxidation mechanism (LOM)^22,23,25^. LOM can bypass the O-O bond formation which was regarded as rate-determining step in AEM and broke up the scaling relationship limitation. Enlightened by the above analysis, the OER mechanism is highly desirable to be implemented, shedding light on the correlation between the mechanism and electrocatalytic performance.

Herein, we report the hierarchical core-shell bimetal nitride/hydroxide (NiMoN/NiFe LDH) heterostructure array with two-dimensional NiFe LDH nanosheets attached to one-dimensional NiMoN nanorods, regulating the electronic states on catalytically active sites and tracking the relationship of structure-activity. As-activated NiMoN/NiFe LDH delivers industrial current density of 1000 mA cm^−2^ at an overpotential of 266 mV and durability of 250 h for OER. Especially, the NiMoN/NiFe LDH forms optimized electronic structure through dynamic structure evolution, recording by in-situ Raman spectroscopy and ultraviolet-visible spectroscopy. The OER kinetics and intermediate evolution of NiMoN/NiFe LDH is exhibited by operando electrochemical impedance spectroscopy (EIS). Moreover, density functional theory (DFT) calculation and differential electrochemical mass spectrometry (DEMS) prove that as-activated NiMoN/NiFe LDH prefers LOM pathway for OER, breaking the scaling relationship limitation and accelerating reaction kinetics. This work paves an avenue to develop efficient heterojunction electrocatalysts for industrial water-splitting electrolysis.

## Results

### Synthesis and structural characterization

**Fig. 1:**
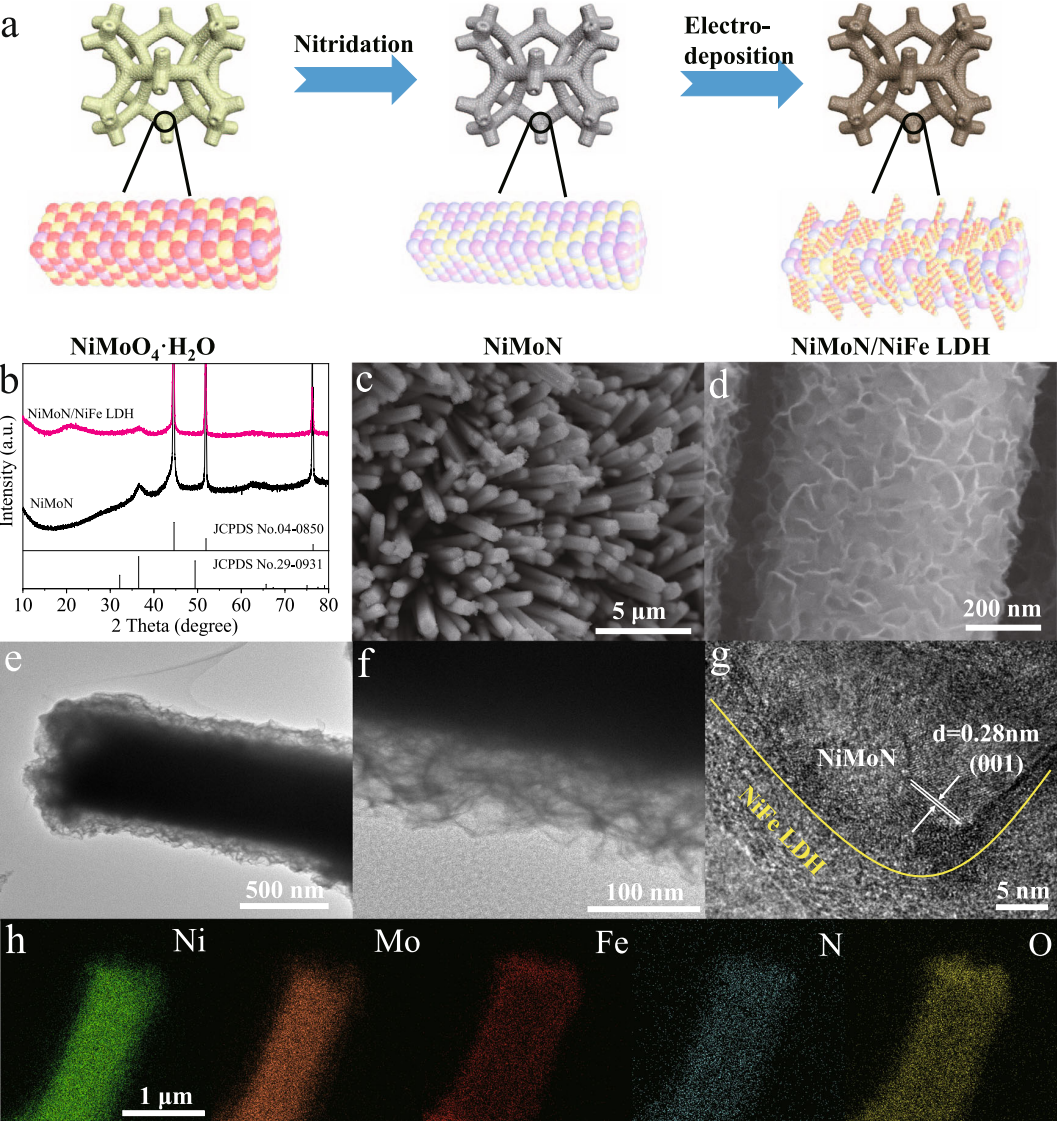
Schematic representation, structural and morphological characterizations. **a** Schematic illustration of the synthesis procedure of NiMoN/NiFe LDH. **b** The XRD patterns of NiMoN and NiMoN/NiFe LDH. **c**, **d** SEM images, **e**, **f** TEM and **g** HR-TEM images of NiMoN/NiFe LDH. **h** EDS element mapping of NiMoN/NiFe LDH.

The synthetic procedure of 3D core-shell NiMoN/NiFe LDH electrocatalyst was illustrated in Fig. 1a, where NiMoN nanorods as the core derived from the NiMoO_4_·H_2_O and amorphous NiFe LDH is used as shell. Briefly, NiMoO_4_·H_2_O nanorods array was perpendicularly grown on the nickel foam (NF) via a facile hydrothermal process^30^. Subsequently, NiMoO_4_·H_2_O nanorods array was calcined in ammonia atmosphere at different temperatures, resulting in Ni_0.2_Mo_0.8_N nanorods array (abbreviated as NiMoN). Finally, the amorphous NiFe LDH nanosheets were electrodeposited on NiMoN nanorods to form 3D hierarchical NiMoN/NiFe LDH electrocatalyst. As-synthesized NiMoN/NiFe LDH heterostructure electrocatalyst not only provides abundant active sites due to multi-interface but also facilitates the mass transfer of reactant and fast release of gas bubbles. X-ray diffraction (XRD) patterns of NiMoN/NiFe LDH are shown in Fig. 1b. The crystalline structure of as-prepared precursor by the first-step hydrothermal reaction can be assigned to NiMoO_4_·H_2_O (Supplementary Fig. 1). After the annealed-treatment in ammonia atmosphere, the diffraction peaks at 36.5° and 65.7° are indexed to (100) and (110) planes of Ni_0.2_Mo_0.8_N (JCPDS No. 29-0931). The remaining three sharp diffraction peaks can be assigned to the substrate of Ni foam. After the electrodeposition process of NiFe LDH nanosheets on NiMoN nanorods (Fig. 1b), no obvious diffraction peak can be detected except for two diffraction peaks from Ni_0.2_Mo_0.8_N, indicating the amorphous and ultrathin feature of NiFe LDH.

To confirm the geometric morphology of hierarchical core-shell NiMoN/NiFe LDH electrocatalyst, scanning electron microscopy (SEM) and transmission electron microscopy (TEM) are employed. As shown in Supplementary Fig. 2, dense NiMoO_4_·H_2_O nanorods array with an average diameter of 0.5 μm and lengths of tens of microns are perpendicularly aligned on NF. After the nitridation treatment of NiMoO_4_·H_2_O nanorods, there is no significant change upon the morphology of NiMoN nanorods except the surface became rougher. Then, NiFe LDH nanosheets were directly electrodeposited on the conductive NiMoN nanorods. As shown in Fig. 1c, d, the ultrathin NiFe LDH nanosheets were vertically and densely distributed on the surface of NiMoN nanorods, indicating the formation of 3D hierarchical architecture of NiMoN/NiFe LDH. In comparison, NiFe LDH nanosheets were directly electrodeposited on NF (Supplementary Fig. 2). Elemental mappings of various arrays were conducted, indicating that these elements were homogeneously distributed (Supplementary Fig. 3~5). The detailed heterostructure of NiMoN/NiFe LDH was further confirmed by TEM images (Fig. 1e, f). It is obvious that NiMoN/NiFe LDH is composed of NiMoN nanorods cores and NiFe LDH nanosheets shells. NiFe LDH is interconnected with each other and tightly grow on NiMoN nanorods, offering abundant active sites and facilitating electrolyte diffusion^31^. The high-resolution TEM (HR-TEM) image shows the heterointerface existed between NiMoN and amorphous NiFe LDH (Fig. 1g and Supplementary Fig. 6). The lattice fringe with a d-spacing of 0.28 nm is attributed to the (001) plane of NiMoN, whereas the shell is dominated by the amorphous NiFe LDH, which is more OER-active than their crystalline counterparts and consistent with the XRD results (Fig. 1b)^32,33^. The different contrast in HR-TEM images is interrelated to the difference in the thickness between the nanosheets and the nanorods. The high-angle annular dark-field scanning TEM (HAADF-STEM) and energy dispersive X-ray (EDX) mapping images showed that Ni, Mo, N, Fe and O elements are distributed in the entire region. (Fig. 1h and Supplementary Fig. 7~8). In comparison, the TEM images of NiMoN nanorods and NiFe LDH nanosheets are shown in Supplementary Fig. 9~10. Therefore, these results demonstrated the 3D hierarchical NiMoN/NiFe LDH electrocatalyst has been synthesized by this typical approach.

**Fig. 2:**
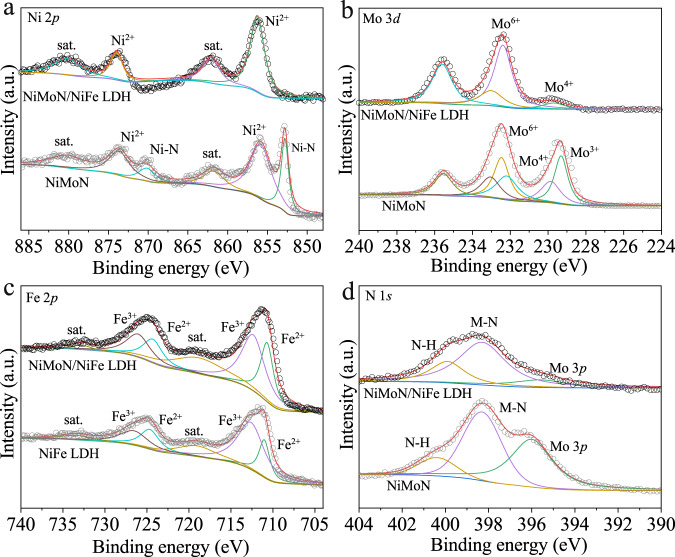
XPS spectra. The high-resolution XPS spectra of **a** Ni 2*p*, **b** Mo 3*d*, **c** Fe 2*p* and **d** N 1*s*.

X-ray photoelectron spectroscopy (XPS) measurement was employed to analyze the surface element composition and chemical state of various electrocatalysts. Ni, Mo, Fe, N and O was identified in the survey spectrum of NiMoN/NiFe LDH (Supplementary Fig. 11). For the Ni 2*p* spectra in Fig. 2a, the binding energy of 852.8, 855.8, 870.1 and 873.6 eV are ascribed to Ni 2*p*_3/2_ and Ni 2*p*_1/2_ of Ni-N and Ni^2+^ for NiMoN nanorods, respectively^33^. The two peaks at 855.6 and 873.4 eV are attributed to Ni^2+^ in NiFe LDH (Supplementary Fig. 12b). After deposition of NiFe LDH, the peaks of Ni-N species disappeared and the two peaks at 856.2 and 873.9 eV can be assigned to divalent Ni from NiMoN/NiFe LDH. The positive shift compared with NiMoN revealed the efficient charge transfer between NiFe LDH and NiMoN^34^. As shown in Fig. 2b, the Mo 3*d* core-level spectra of NiMoN can be well-deconvoluted into three doublets at 229.3, 229.8, 232.3, 232.5, 233.0 and 235.5 eV, which can be attributed to Mo 3*d*_5/2_ and Mo 3*d*_3/2_ of Mo^3+^, Mo^4+^ and Mo^6+^, respectively^16,33^. After the decoration of NiFe LDH, there remain two valence states of Mo^4+^ and Mo^6+^ in Mo 3*d* spectrum from NiMoN/NiFe LDH. The Fe 2*p* XPS spectra of NiMoN/NiFe LDH in Fig. 2c shows four deconvoluted peaks at 710.9, 712.5, 724.5 and 726.3 eV, corresponding to Fe 2*p*_3/2_ and Fe 2*p*_1/2_ of Fe^2+^ and Fe^3+^, respectively, which is negative shift in binding energy compared with NiFe LDH, indicating the strong electronic interactions between NiFe LDH and NiMoN^32^. The N 1 *s* spectra of NiMoN shows three peaks at 395.6, 398.3 and 399.9 eV, attributing to Mo 3*p*_3/2_, Ni/Mo-N and N-H moieties (Fig. 2d), in which the latter results from incomplete reaction with ammonia^18,35^. The binding energy of NiMoN/NiFe LDH is similar with NiMoN, along with the decreased strength due to the deposition of the NiFe LDH. From the high-resolution O 1*s* spectra of NiMoN/NiFe LDH, the peaks at 529.6, 531.0 and 532.4 eV can be attributed to metal-oxygen bond, hydroxyl species and adsorbed water molecules on the surface (Supplementary Fig. 11b)^36^. The XPS spectra of NiFe LDH and NiMoO·H_2_O were conducted (Supplementary Fig. 12~13). Based on the above analysis, the strong heterogeneous interactions between NiFe LDH and NiMoN modulate the surface electronic structure, possibly enhancing electrocatalytic activity.

### Electrocatalytic performance for OER

**Fig. 3:**
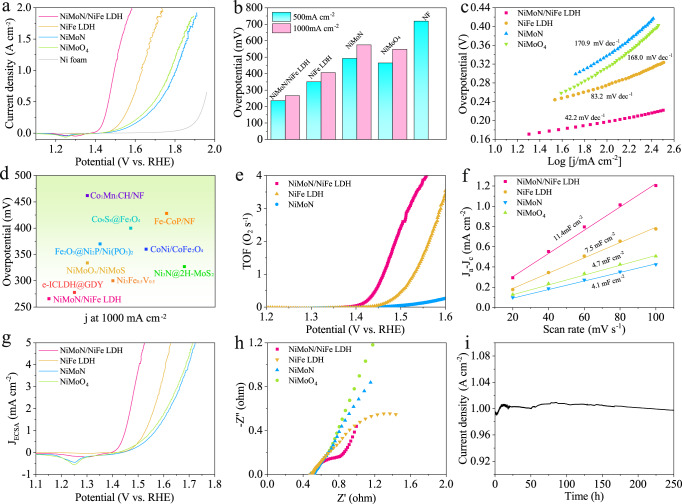
OER catalytic performance. **a** The OER polarization curves of as-activated NiMoN/NiFe LDH, NiFe LDH, NiMoN, NiMoO_4_ and Ni foam in 1 M KOH. **b** The overpotential of catalysts at different current densities. **c** Corresponding Tafel slope. **d** Comparison of the overpotentials for reported electrocatalysts at 1000 mA cm^−2^. **e** The potential-dependent TOF plot of as-activated NiMoN/NiFe LDH, NiFe LDH and NiMoN. **f** Double-layer capacitance (C_dl_). **g** ECSA normalized polarization curves. **h** Electrochemical impedance spectroscopy. **i** The chronoamperometry curve of as-activated NiMoN/NiFe LDH for a continuous 250 h operation at a constant potential.

The electrocatalytic OER performance of various catalysts was evaluated by a standard three-electrode setup in O_2_-saturated alkaline solution and the potentials were calibrated versus reversible hydrogen electrode (vs. RHE). Figure 3a shows the *iR*-corrected linear sweep voltammetry (LSV) polarization curves of various electrocatalysts, exhibiting the highest OER performance of as-activated NiMoN/NiFe LDH. Strikingly, as-activated NiMoN/NiFe LDH requires the overpotentials of only 236 and 266 mV at the current densities of 500 and 1000 mA cm^−2^, dramatically lower than those of NiFe LDH (351 and 406 mV), NiMoN (492 and 575 mV), NiMoO_4_ (465 and 548 mV), and Ni foam (719 mV) in Fig. 3b. Moreover, the value surpasses most heterostructure catalysts in the previous reports (Fig. 3d and Supplementary Table 1)^37–40^. As shown in Fig. 3c, as-activated NiMoN/NiFe LDH possess the Tafel slope of 42.2 mV dec^-1^, which is lower than 83.2 mV dec^-1^ for NiFe LDH and 170.9 mV dec^-1^ for NiMoN, indicating the rapid reaction kinetics toward electrocatalytic water oxidation. To gain a general understanding of the intrinsic activity of as-activated NiMoN/NiFe LDH, turnover frequency (TOF) and electrochemical surface area (ECSA) normalized current density have been estimated. The number of active sites was quantified through an electrochemical method according to the literature^41^. The TOF of as-activated NiMoN/NiFe LDH is 3.39 s^−1^ at 1.53 V vs. RHE, which is ~3 times and ~37 times higher than those of NiFe LDH (1.03 s^−1^) and NiMoN (0.09 s^−1^), respectively (Fig. 3e and Supplementary Fig. 14). The ECSA value was measured through the electrochemical double-layer capacitances (C_dl_) determined by cyclic voltammetry method. As-activated NiMoN/NiFe LDH possesses the highest C_dl_ value of 11.4 mF cm^−2^, which is higher than those of NiFe LDH (7.5 mF cm^−2^) and NiMoN (4.1 mF cm^−2^), indicating that more exposed catalytic active sites are attained by the hierarchical architecture (Fig. 3f and Supplementary Fig. 15). In Fig. 3g, ECSA-normalized current density has the same trend as current density evaluated by geometric surface area, indicating the improvement of electrocatalytic activity is ascribed to the enlarged ECSA and the promoted intrinsic activity^42^. The larger TOF value and higher ECSA-normalized current density of as-activated NiMoN/NiFe LDH as compared to other samples reveal that the heterostructure plays a significant role in facilitating kinetics toward water oxidation. EIS was measured to detect the charge-transfer property. As shown in Fig. 3h, the Nyquist plot is fitted using Randles equivalent circuit model. As-activated NiMoN/NiFe LDH possesses a smaller semicircle, presenting the excellent conductivity and rapid electronic transport of the heterostructure. Long-term stability is a pivotal parameter to estimate the electrocatalytic performance, especially at large current density and large-scale water splitting application. As shown in Fig. 3i, as-activated NiMoN/NiFe LDH was observed by the continuous test over 250 h at a constant potential without obvious decay in current density around 1000 mA cm^−2^, suggesting the excellent catalytic stability of the catalyst for water oxidation. The Faradaic efficiency (FE) was calculated in comparison to the amount oxygen produced experimentally against theoretical quantity. The FE of as-activated NiMoN/NiFe LDH is about 98.6% (Supplementary Fig. 16), indicating no side reaction occurred during OER process.

A series of control experiments were conducted for NiMoN/NiFe LDH. Firstly, the effect of nitridation temperature on the preparation of NiMoN-T/NiFe LDH (T represents the nitridation temperature) was investigated. From XRD pattern (Supplementary Fig. 17), the crystallinity of NiMoN improved with the increasing nitridation temperature. The OER performance of NiMoN under different nitridation conditions and the decoration of NiFe LDH nanosheets has been measured. As presented in Supplementary Fig. 18, as-activated NiMoN/NiFe LDH drives the current density of 500 mA cm^−2^ at the overpotential of 236 mV, which is smaller than that of NiMoN-400/NiFe LDH (275 mV) and NiMoN-600/NiFe LDH (286 mV), indicating that the best nitridation condition is 500 °C. The effect of different calcination atmosphere on NiMoO_4_·H_2_O was also investigated. MoNi_4_/MoO_2_ and NiMoO_4_ were formed in a reductive and argon atmosphere, respectively (Supplementary Fig. 19). For a better comparison, NiFe LDH nanosheets were also deposited on MoNi_4_/MoO_2_ and NiMoO_4_. The MoNi_4_/MoO_2_/NiFe LDH and NiMoO_4_/NiFe LDH requires the overpotentials of 271 and 330 mV at 500 mA cm^−2^, which is worse than that of NiMoN nanorods (Supplementary Fig. 20). This result indicated that the nitridation treatment is better choice due to the good electrical conductivity for these catalysts.

The electrochemical HER performance of NiMoN/NiFe LDH was assessed in N_2_-saturated 1 M KOH, corresponding to the LSV polarization curves with *iR* compensation (Supplementary Fig. 21a). To deliver the current densities of 500 and 1000 mA cm^−2^, NiMoN/NiFe LDH presents the smallest overpotentials of 150 and 205 mV among the electrocatalysts including NiMoN (210 and 260 mV), NiFe LDH (455 and 509 mV), NiMoO_4_ (482 and 530 mV), and Ni foam (588 and 711 mV), demonstrating the substantial improvement in catalytic activity after the decoration of NiFe LDH nanosheets (Supplementary Fig. 21b). The Tafel slope of NiMoN/NiFe LDH is 39.1 mV dec^−1^, indicating the rapid reaction kinetics and the reaction process follows the Volmer-Heyrovsky mechanism with the Heyrovsky as the rate-determining step (Supplementary Fig. 21c). The synergistic effect of the heterostructure could enhance the HER activity. NiFe LDH nanosheets as the shell promote the water dissociation and enhance the rate for the formation of adsorbed hydrogen (H_ad_) intermediates. Subsequently, H_ad_ transfers and adsorbs on the NiMoN nanorods to combine with another H_ad_ or adsorbed water molecule to form H_2_^14,43,44^. Furthermore, EIS was performed to probe the charge-transfer kinetics. NiMoN/NiFe LDH shows the smallest semicircle diameter (Supplementary Fig. 21d), revealing a rapid catalytic kinetics electron-transfer process. The calculated C_dl_ value of NiMoN/NiFe LDH from CV scans with different rates is 17.5 mF cm^−2^, which is the largest among as-obtained electrocatalysts, indicating more exposure area of the active sites attained by hierarchical architecture (Supplementary Fig. 22~23). The I-t curves were used to evaluate the long-term stability of the catalysts at a constant potential. NiMoN/NiFe LDH exhibits negligible degradation in 100 h (Supplementary Fig. 24), indicating superior catalytic stability and promising of industrial application.

### Electrocatalytic performance for overall water splitting

**Fig. 4:**
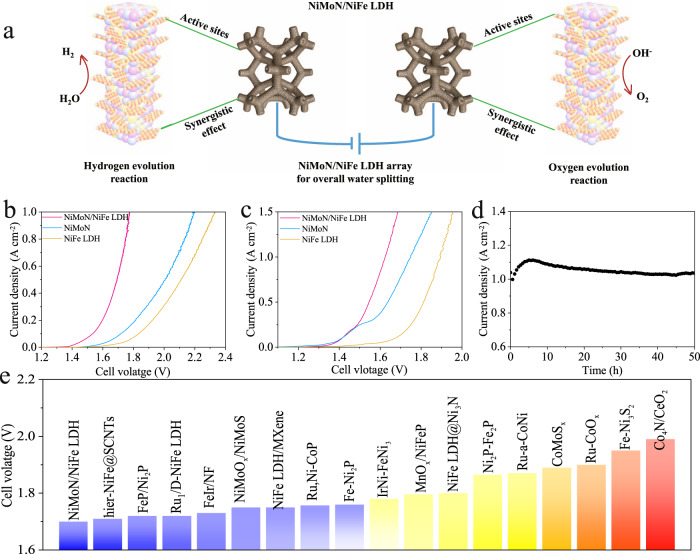
Schematic diagram and catalytic performance of overall water splitting. **a** Schematic diagram of overall water splitting in a two-electrode system. Overall water splitting performance of as-activated NiMoN/NiFe LDH in **b** 1 M KOH at 25 °C and **c** 30% KOH at 80 °C. **d** Chronoamperometry curve of as-activated NiMoN/NiFe LDH toward overall water splitting in 30% KOH at 80 °C. **e** Comparison of the cell voltage with as-activated NiMoN/NiFe LDH and other electrocatalysts at current density of 500 mA cm^−2^.

Considering the excellent HER and OER electrochemical activities, as-activated NiMoN/NiFe LDH served as bifunctional catalysts for both anode and cathode to assemble a two-electrode system for overall water splitting (Fig. 4a). As-activated NiMoN/NiFe LDH | | NiMoN/NiFe LDH exhibits the low cell voltages of 1.70 and 1.77 V at the current densities of 500 and 1000 mA cm^−2^ in 1 M KOH at 25 °C (Fig. 4b). We note that performance of NiMoN/NiFe LDH outperforms NiMoN (2.00 and 2.20 V), NiFe LDH (2.11 and 2.33 V), and other electrocatalysts reported in previous literature, highlighting the potential for practical overall water splitting. (Fig. 4e and Supplementary Table 2)^37,39^. Furthermore, the industrial conditions are implemented to seek the potential for the large-scale application. Strikingly, NiMoN/NiFe LDH delivers the current densities of 500 and 1000 mA cm^−2^ as low as 1.54 and 1.62 V in 30% KOH at 80 °C (Fig. 4c). Concerning the stability as a significant parameter, the electrocatalyst should operate over a long-term test under large current density. The two-electrode system shows no evident fluctuation at a constant potential for a current density of 1000 mA cm^−2^ for 50 h, suggesting good durability (Fig. 4d). Alkaline anion exchange membrane (AEM) water electrolysis is a competitive way to produce clean hydrogen fuel. Furthermore, as-activated NiMoN/NiFe LDH was integrated into a membrane electrode assembly (MEA) to evaluate the water splitting performance at different temperature (Supplementary Notes 1 and Fig. 25). As-activated NiMoN/NiFe LDH achieves the industrial current density of 1000 mA cm^−2^ at the cell voltage of 2.29 V at 25 °C. With the temperature increase to 60 °C, the electrocatalyst requires the cell voltage of 1.85 and 1.92 V to drive the current densities 500 and 1000 mA cm^−2^, which was lower than that at 25 °C, indicating the activities of the electrocatalysts are related to the operating temperature. In brief, the 3D hierarchical core-shell structure of NiMoN/NiFe LDH leads to the electron redistribution at the interface and modulates the electronic structure, facilitating the water dissociation and adjusting the binding strength of adsorbed intermediates. Secondly, the combination of NiMoN nanorods and NiFe LDH nanosheets not only gives a large surface area and more exposed active sites, but also improves the intrinsic activity of catalyst. Moreover, NiMoN/NiFe LDH nanoarray was in situ grown on a current collect without use of polymer binder. The nanoarrays accelerates mass transfer, ensures sufficient contact with the electrolyte, and facilitates the release of bubbles. All these advantages promote the catalytic activity of as-activated NiMoN/NiFe LDH and offer an opportunity to practical application.

### In-situ spectroelectrochemistry analysis

**Fig. 5:**
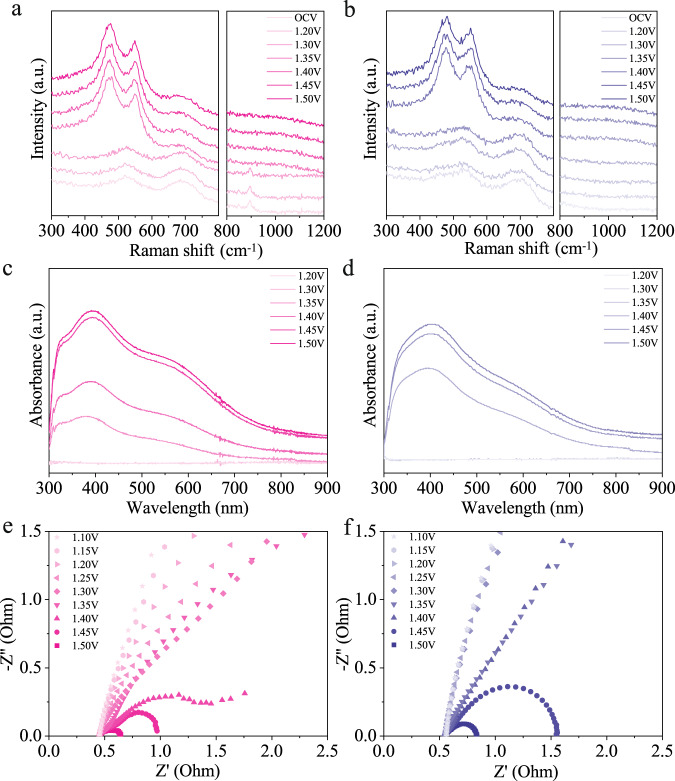
Mechanism analysis. In-situ Raman spectroscopy of **a** NiMoN/NiFe LDH and **b** NiFe LDH at various potentials. In-situ UV-Vis absorption spectra of **c** NiMoN/NiFe LDH and **d** NiFe LDH at various potentials. Nyquist plots for **e** NiMoN/NiFe LDH and **f** NiFe LDH at different applied potentials.

To identify the active phase and dynamic surface reconstruction of NiMoN/NiFe LDH, in-situ Raman spectroscopy was performed and acquired as the function of applied potential with an interval of 0.1 V. At the open circuit potential (OCP), NiMoN/NiFe LDH and NiFe LDH show characteristic peaks at 532 and 702 cm^−1^, which can be attributed to Ni-O in disordered Ni(OH)_2_ and Fe-O in *γ*-FeOOH^45,46^, respectively (Fig. 5a, b). Specifically, a well-defined peak at 890 cm^−1^ is assigned to Mo-O bond for NiMoN/NiFe LDH. For NiMoN/NiFe LDH, two characteristic peaks appeared at 473 and 551 cm^−1^ beyond 1.35 V, corresponding to *E*_g_ bending and *A*_1g_ stretching vibration of Ni^III^-O in *γ*-NiOOH, respectively. While Mo-O bond at 890 cm^−1^ has vanished at this potential^46^. The appearance of these two peaks indicated that *γ*-Ni(Fe)OOH is the active species for NiMoN/NiFe LDH in OER process. Moreover, a broad band in the region of 900~1100 cm^−1^ assigned to *ν*(O-O) of active oxygen species was observed above 1.35 V, which is generated by the deprotonation of oxyhydroxide^47^. Similar Raman peaks appeared until the potential up to 1.4 V for NiFe LDH. In addition, the higher *I*_473_/*I*_551_ ratio of NiMoN/NiFe LDH than NiFe LDH at 1.5 V should be assigned to *γ*-NiOOH, revealing the approximate oxidation state of +3.5^48^. Thus, the generation of active species is easier and more active Ni^3+/4+^ species accumulate for as-activated NiMoN/NiFe LDH, promoting the acquisition of optimized electronic structure for OER.

In-situ ultraviolet-visible (UV-Vis) spectroelectrochemistry tests were conducted in home-made cell to get insight into metal redox process during OER. As shown in Fig. 5c, d, a conspicuous spectroscopic feature was observed for NiMoN/NiFe LDH and NiFe LDH at different applied potentials. There is a broad absorption band between 350 and 600 nm with increasing anodic potential, suggesting the oxidation of Ni center, which is assigned to nickel d-d interband transition and the formation of active oxygen species^49,50^. The accumulation of oxidized species for NiMoN/NiFe LDH starts at 1.35 V vs. RHE, which is in a lower potential compared with NiFe LDH, indicating the heterostructure is beneficial to generate the active species. The absorbance intensity was recorded through potential cycling to track the variation for the oxidation state of Ni (Supplementary Fig. 26). The absorbance intensity rises along with the Ni redox wave. NiMoN/NiFe LDH shows the increased absorption intensity with an onset potential shift to more negative potentials than NiFe LDH, demonstrating the heterointerface enables to generate higher oxidation state of Ni and improves the reaction kinetics, in accordance with the analysis of in-situ Raman spectra.

EIS is a useful electrochemical measurement to probe the properties of electrode/electrolyte interfaces and the adsorption kinetics of reactants on the electrode surface. Operando EIS was performed to get in-depth information on electrochemical reaction kinetics. Figure 5e, f shows the Nyquist plot of NiMoN/NiFe LDH and NiFe LDH in 1 M KOH from 1.10 to 1.50 V. The total resistance of as-activated NiMoN/NiFe LDH and NiFe LDH at different applied potentials are quantified from Nyquist plots (Supplementary Fig. 27a). It is obvious that NiMoN/NiFe LDH presents smaller charge transfer resistance within the range of applied potential, indicating the heterostructure accelerates interfacial charge transfer, which could promote surface activation of electrocatalyst^51^. Moreover, the evolution of reactants (*OH) on the catalysts surface could be described by total resistance. The resistance of NiMoN/NiFe LDH was much lower than that of NiFe LDH within 1.30 V, revealing the faster kinetics for adsorption of *OH at low driving potential. The pseudocapacitance arising from *OH was defined as C_φ_, which was utilized to quantify the adsorption coverage of *OH^52^. The C_φ_ of NiMoN/NiFe LDH is higher than that of NiFe LDH in the whole potential, indicating the higher coverage of *OH for NiMoN/NiFe LDH (Supplementary Fig. 27b, 28). The fast *OH accumulation of NiMoN/NiFe LDH should be in favor of overall catalytic driving force^53,54^. Moreover, the evolution of adsorbed *OH on electrocatalysts was evaluated based on Laviron equation^54^. The steady redox currents all exhibit linear correlation with the square root of potential scan rate in CV (5~700 mV s^−1^). The *K*_s_ value of NiMoN/NiFe LDH is 0.14 s^−1^, which is larger than that of NiFe LDH (0.11 s^−1^), revealing the strong binding strength of *OH (Supplementary Fig. 29~31). Moreover, the Bode plot reflects both the dynamic evolution of electrocatalysts and OER process, exhibiting the variation of phase angle with frequency. Generally, the peaks of phase angle at low and high frequency are on account of surface charge conduct and electron transfer in inner layer of catalyst, respectively, corresponding to the OER process and electrocatalyst electrooxidation reaction^51^. At the potential of 1.35 V, the phase angle of NiMoN/NiFe LDH reduces much quicker at the high-frequency region, indicating the violent electrocatalyst electrooxidation reaction with the fast charge transfer of electrocatalyst inner-layer (Supplementary Fig. 32). While NiFe LDH experiences similar phenomenon at more positive potential, consistent with in-situ Raman and UV-Vis spectra. In the low-frequency region, the smaller phase angle reveals that OER process occurs drastically after 1.40 V for NiMoN/NiFe LDH. For the NiFe LDH, OER process starts at 1.45 V. In a word, NiMoN/NiFe LDH has a faster charge transfer in surface and inner layer, revealing a better electrochemical performance compared with NiFe LDH. This is attributed to the ensemble effect and electronic interaction, thus optimizing adsorption energies and accelerating reaction kinetics.

As-activated NiMoN/NiFe LDH electrocatalyst after OER test was characterized to check the crystal structure and chemical state. From XRD patterns, the peak intensity weakened after the OER test (Supplementary Fig. 33). The TEM image shows that as-activated NiMoN/NiFe LDH retains the core-shell structure and NiFe LDH nanosheet attached NiMoN nanorods tightly, consistent with SEM results (Supplementary Fig. 34). For the high-resolution XPS spectra of Ni 2*p*, the binding energy positively shifts and the higher valence states of Ni^3+^ appears, indicating the formation of oxyhydroxides. Besides, the main peak of Fe 2*p* positively shifts towards higher binding energy compared with pristine electrocatalyst, demonstrating the oxidation state of Fe (+3). Moreover, the signals of Mo 3*d* and N 1*s* after OER test were weaker than the pristine catalyst (Supplementary Fig. 35).

### Identification of mechanism

**Fig. 6:**
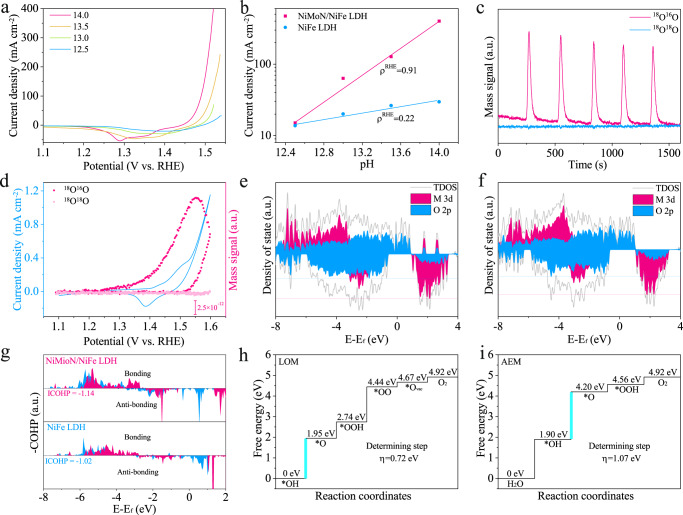
Mechanism and theoretical analysis. **a** Linear sweep voltammetry curve of as-activated NiMoN/NiFe LDH in alkaline electrolytes with different pH. **b** The logarithms of current density at 1.50 V vs. RHE against the pH. The DEMS signals of ^34^O_2_ and ^36^O_2_ vs. **c** time and **d** applied potential for as-activated NiMoN/NiFe LDH. The calculated DOS of metal 3d, oxygen 2p and total DOS for **e** NiMoN/NiFe LDH and **f** NiFe LDH. **g** Calculated crystal orbital Hamilton population (COHP). Gibbs free energy diagram of OER steps for **h** LOM pathway and **i** AEM pathway.

To get deep insight into the reaction mechanism, a series of electrocatalytic measurements were performed. The catalytic performance of as-activated NiMoN/NiFe LDH in alkaline electrolytes with increasing pH values from 12.5 to 14 was shown in Fig. 6a, b to detect the proton-electron transfer kinetics. The activity of as-activated NiMoN/NiFe LDH exhibits strong pH dependence, revealing the non-concerted proton-electron transfer process^22,23^. While NiFe LDH showed significantly lower pH-dependent OER kinetics with dominant CPET steps (Supplementary Fig. 36). Therefore, we proposed as-activated NiMoN/NiFe LDH follows the LOM pathway rather than AEM pathway, in which lattice oxygen directly participates in OER process. To direct clarify the lattice oxygen oxidation process, ^18^O isotope labeling DEMS was conducted by NiMoN/NiFe LDH. Firstly, NiMoN/NiFe LDH was electrochemically activated in 0.1 M KOH electrolyte with H_2_^18^O and rinsed by H_2_^16^O after labeled process. Afterward, the ^18^O isotope labeled catalysts were tested in 0.1 M KOH with H_2_^16^O by CV test, and the generated gaseous product was monitored by mass spectrometry. In Fig. 6c, the peak of ^18^O^16^O (mass-to-charge, m/z = 34) with pronounced periodical intensity was observed for as-activated NiMoN/NiFe LDH, while no signal for ^18^O^18^O (m/z = 36). The results testified lattice oxygen was involved in OER process and half of oxygen atom in oxygen was derived from lattice oxygen, while another oxygen was from the electrolyte^22,25^. The mass signal of ^18^O^16^O was plotted against the anodic potential, and it shows similar variation tendency under CV measurement, revealing the dynamic trace of DEMS (Fig. 6d). Moreover, NiFe LDH exhibits similar periodic signal of ^18^O^16^O and the much lower intensity reveals that the degree of lattice oxygen involvement is lower (Supplementary Fig. 37~38). Above results indicates as-activated NiMoN/NiFe LDH prefers LOM pathway.

To gain theoretical insights into the transformation of the reaction mechanism, DFT calculations were employed to determine the electronic structure and energy barrier (Supplementary Notes 2). Based on the experiment results, NiFeOOH and Mo-doped NiFeOOH were selected as model for DFT calculation. Firstly, the projected density of state (PDOS) was employed to analyze the orbital distribution of metal d band and oxygen p band for both NiMoN/NiFe LDH and NiFe LDH (Fig. 6e, f). The overlap of metal d band and O 2p bands evidently, reveals the covalent hybridization of metal sites and oxygen ligands enhanced^23^. Moreover, the electronic configuration and metal-oxygen bond strength were evaluated by crystal orbital Hamilton populations (COHP). The higher occupied anti-bonding states of Ni 3d band near Fermi level for NiMoN/NiFe LDH manifests the stronger hybridization between the metal d band and O 2p band. The metal-oxygen bond strength was quantified by the integral of COHP up to Fermi level, and the larger absolute value of ICOHP of Ni-O (−1.14) bonding for NiMoN/NiFe LDH reveals the enhanced covalency^26^. The enhanced covalency of M-O bond promotes the delocalization of electrons in NiMoN/NiFe LDH, providing a premise for the participation of lattice oxygen during OER. Based on the above analysis, OER process through different reaction pathway was simulated by DFT calculation. For OER process based on LOM pathway, the catalyst experiences deprotonation to form exposed lattice oxygen (Supplementary Fig. 39). Then, the OH^-^ adsorbs on lattice oxygen by nucleophilic attacking. After the deprotonation of *OOH, the oxygen releases and leave oxygen vacancy sites on the surface. In the end, the produced oxygen vacancy site is refilled by OH^-^. The calculated Gibbs free energy for LOM was implemented (Fig. 6h)^54,55^. For NiMoN/NiFe LDH, the deprotonation of *OH was the rate-determine step (RDS) with the overpotential of 0.72 eV. As for the conventional AEM pathway, it undergoes four CPET steps with the oxygen intermediates (*OH, *O and *OOH) on metal sites (Supplementary Fig. 40). The RDS was the conversion from *OH to *O intermediates with a high energy barrier of 2.29 eV (Fig. 6i). This consequence is consistent with the results of ^18^O labeling DEMS, indicating the reaction mechanism was switched to LOM.

## Discussion

In summary, the hierarchical heterostructure electrocatalysts of NiMoN/NiFe LDH have been synthesized by attaching NiFe LDH nanosheets on NiMoN nanorods, regulating the electronic states on catalytically active sites and tracking the relationship of structure-activity. Remarkably, as-activated NiMoN/NiFe LDH exhibits a low overpotential of 266 mV to deliver an industrial current density of 1000 mA cm^−2^, maintaining decent performance for at least 250 h for OER. The as-activated NiMoN/NiFe LDH promotes the generation of high valence active sites to optimize the electronic structure and accelerate OER kinetics by in-situ spectroelectrochemistry. From both theoretical and experimental results, the metal-oxygen covalency is enhanced and the OER mechanism switched to lattice oxygen mechanism. This work opens an avenue for the rational design of nonprecious based and efficient electrocatalysts for sustainable hydrogen production from industrial water splitting.

## Methods

### Synthesis of NiMoN nanorods

Firstly, a piece of nickel foam (2 × 3 cm^2^) was immersed in the solution containing 0.04 M Ni(NO_3_)_2_·6H_2_O and 0.01 M (NH_4_)_6_Mo_7_O_24_·4H_2_O with 15 ml H_2_O and transferred into a 25 mL Teflon vessel. Then, the vessel was sealed in a stainless autoclave and heated to 150 °C and kept for 6 h. After cooling down to room temperature, the NiMoO_4_·H_2_O washed with DI water and ethanol and dried in an oven at 60 °C. Finally, the as-prepared NiMoO_4_·H_2_O nanorods were heated to 500 °C at a ramp rate of 5 °C/min and maintained for 2 h in NH_3_ atmosphere. After the furnace naturally cooled down to room temperature and NiMoN nanorods were obtained.

### Synthesis of NiMoN/NiFe LDH

The NiFe LDH nanosheets were electrodeposited on the surface of NiMoN nanorods to obtain NiMoN/NiFe LDH. The electrodeposition was conducted in a standard three-electrode setup, using NiMoN as working electrode, a parallel platinum net as counter electrode and Ag/AgCl(saturated) as reference electrode. The electrolyte was containing 0.06 M Ni(NO_3_)_2_·6H_2_O and 0.048 M Fe(NO_3_)_3_·9H_2_O. The electrodeposition potential was −1.0 V vs. Ag/AgCl for 200 s. Then, the as-synthesized electrode was rinsed with deionized water and ethanol and dried at 60 °C. The loading weight of the formed electrocatalysts on the nickel foam was ≈ 15.8 mg cm^2^.

### Synthesis of NiFe LDH

The NiFe LDH nanosheet was direct electrodeposited on NF as working electrode under the same procedure for NiMoN/NiFe LDH.

### Structural characterization

X-ray diffraction patterns were characterized using X-ray diffractometer (Rigaku Rotaflex, Japan) by Cu K_α_ radiation (λ = 1.5418 Å). Field emission scanning electron microscope (FE-SEM) tests were recorded on HITACHI SU5000 with an accelerating voltage of 10 kV and energy-dispersive X-ray spectrum (EDS). Transmission electron microscope (TEM) with high-resolution mode and EDS elemental mapping were performed on FEI Tecnai F30 at an accelerating voltage of 200 kV. X-ray photon energy spectroscopy (XPS) was performed using a Thermo Fisher ESCALAB 250Xi. In-situ Raman experiments were measured by a Raman spectrometer (Thermo Fisher, DXR Microscope) and the laser wavelength is 532 nm. The UV-Vis measurements were conducted with UV-Vis-NIR spectrophotometer (Shimadzu UV-3600 Plus). The in-situ UV-Vis experiments were performed with a home-made quartz cuvette. The catalyst-loaded fluorine-doped tin oxide (FTO) glass was used as working electrode and the electrocatalysts loading was 0.1 mg cm^2^.

### Electrochemical measurements

The electrochemical measurements were conducted with Corrtest CS310M using a standard three-electrode cell. As-synthesized electrode was employed as the working electrode. A Pt wire and Hg/HgO electrode were applied as the counter electrode and reference electrode, respectively. All potentials were converted to RHE using the following equation: *E*_RHE_ = *E*_Hg/HgO_ + 0.059 pH + 0.098 V. The working area is 1×1 cm^2^. The LSV polarization curves were recorded with a sweeping rate of 2 mV s^−1^ in oxygen-saturated 1 M KOH at 25 °C. The 85% *iR* compensation was performed manually for polarization curves after the measurement for ohmic resistance by EIS. The ECSA was measured through a series of cyclic voltammetry at various scan rate in the non-Faradaic region. EIS was performed over the frequency range from 100 kHz to 0.01 Hz at the potential 1.48 V vs. RHE by applying an AC voltage of 5 mV amplitude. For the measurement of FE, gas products were measured by gas chromatography (Shimadzu, GC-2014), where produced oxygen was collected by online sampling system.

### First-principle calculation

DFT calculations were carried out using the Vienna Ab-initio Simulation Package (VASP)^56^. The Perdew-Burke-Ernzerhof (PBE) functional within the generalized gradient approximation (GGA) was used to describe the exchange-correlation interactions. The electron-ion interactions were described by projector augmented wave (PAW) potentials^57^. A kinetics energy cut-off of 450 eV was used. The force and energy convergence criteria were set as 0.02 eV Å^−1^ and 10^−4^ eV. The Brillouin zone was sampled with a 3×3×1 Monkhorst-Pack grid. The DFT-D3 method was used to evaluate the van der Waals (vdW) correction^58^. The Hubbard-U terms for Ni and Fe were considered, with the effective U value of 4.0 and 4.3 eV for Ni and Fe, respectively. The water solvation effect was also considered by using VASPsol^59^. The COHP of considered atomic pairs was calculated by the Lobster code^60^.

## Supplementary information

Supplementary Information

## Data Availability

The data reported in this paper are available from the corresponding author upon reasonable request.
