# Supplementary material for: Regulating electronic states of nitride/hydroxide to accelerate kinetics for oxygen evolution at large current density
Source: Nat Commun. 2023 Apr 4;14:1873. doi: 10.1038/s41467-023-37091-x (PMC10073178; doi:10.1038/s41467-023-37091-x)
Supplement: Supplementary file 1 — Supplementary Information [file 41467_2023_37091_MOESM1_ESM.pdf]

Supporting Information

Regulating electronic states of nitride/hydroxide to accelerate kinetics for oxygen evolution at large current density

Panlong Zhai[1,5], Chen Wang[1,5], Yuanyuan Zhao[2,5], Yanxue Zhang[2], Junfeng Gao[2*], Licheng Sun[3,4] and Jungang Hou[1*]

[1]State Key Laboratory of Fine Chemical, Frontiers Science Center for Smart Materials Oriented Chemical Engineering, School of Chemical Engineering, Dalian University of Technology, Dalian 116024, P. R. China

[2]State Key Laboratory of Structural Analysis, Optimization and CAE Software for Industrial Equipment, Dalian University of Technology, Dalian 116024, P. R. China

[3]Center of Artificial Photosynthesis for Solar Fuels and Department of Chemistry, School of Science, Westlake University, Hangzhou 310024, P. R. China

[4]Department of Chemistry, School of Engineering Science in Chemical, Biotechnology and Health KTH Royal Institute of Technology, Stockholm 10044, Sweden

[5]These authors contributed equally: Panlong Zhai, Chen Wang and Yuanyuan Zhao.

Corresponding author: Prof. Junfeng Gao and Prof. Jungang Hou

Email: gaojf@dlut.edu.cn; jhou@dlut.edu.cn

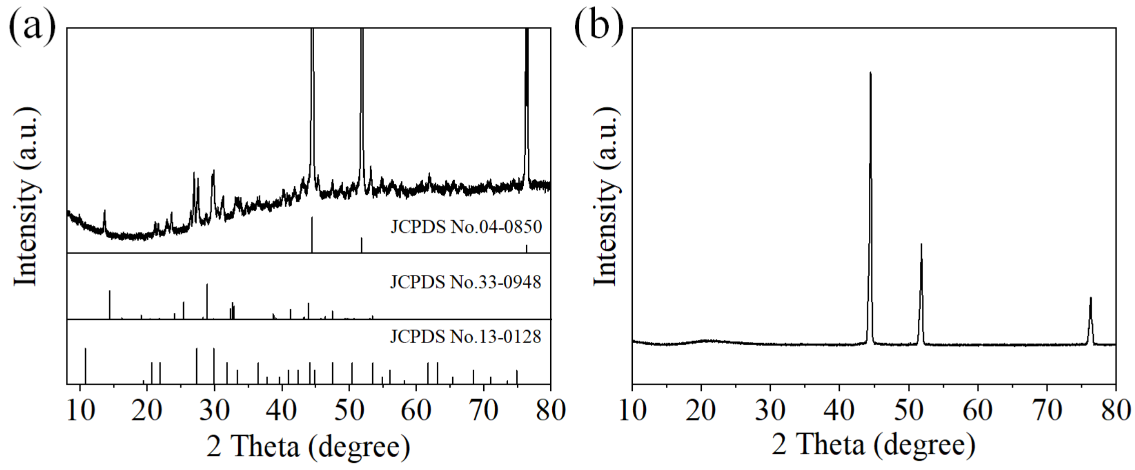

Supplementary Fig. 1. (a) The XRD pattern of $NiMoO_4 \cdot H_2O$ nanorods. The expected reflections of metallic Ni (JCPDS No. 04-0850), $NiMoO_4$ (JCPDS No. 33-0948) and $NiMoO_4 \cdot H_2O$ (JCPDS No. 13-0128) are shown in the bottom line. (b) The XRD pattern of NiFe LDH nanosheets.

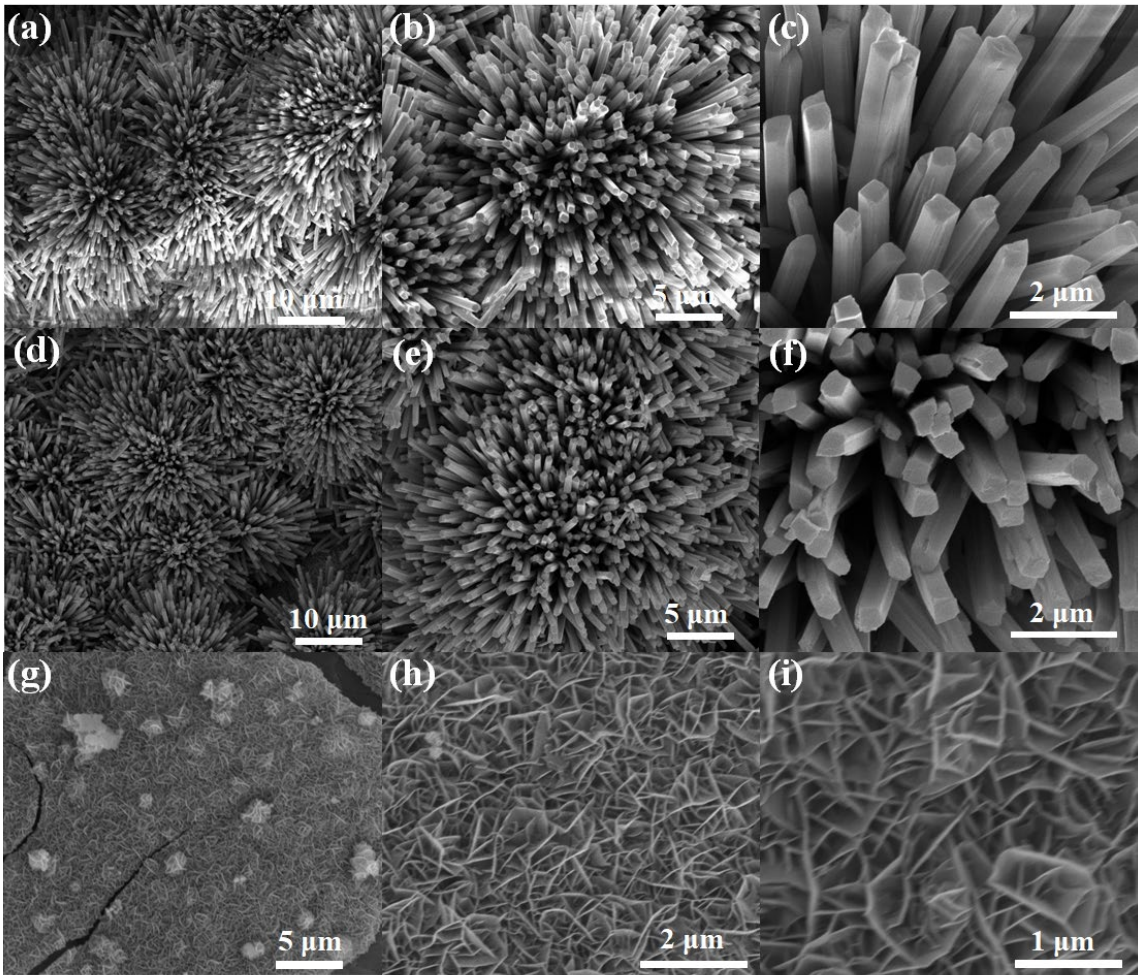

Supplementary Fig. 2. The SEM images of (a-c) $NiMoO_4 \cdot H_2O$ nanorods, (d-f) NiMoN nanorods and (g-i) NiFe LDH nanosheets.

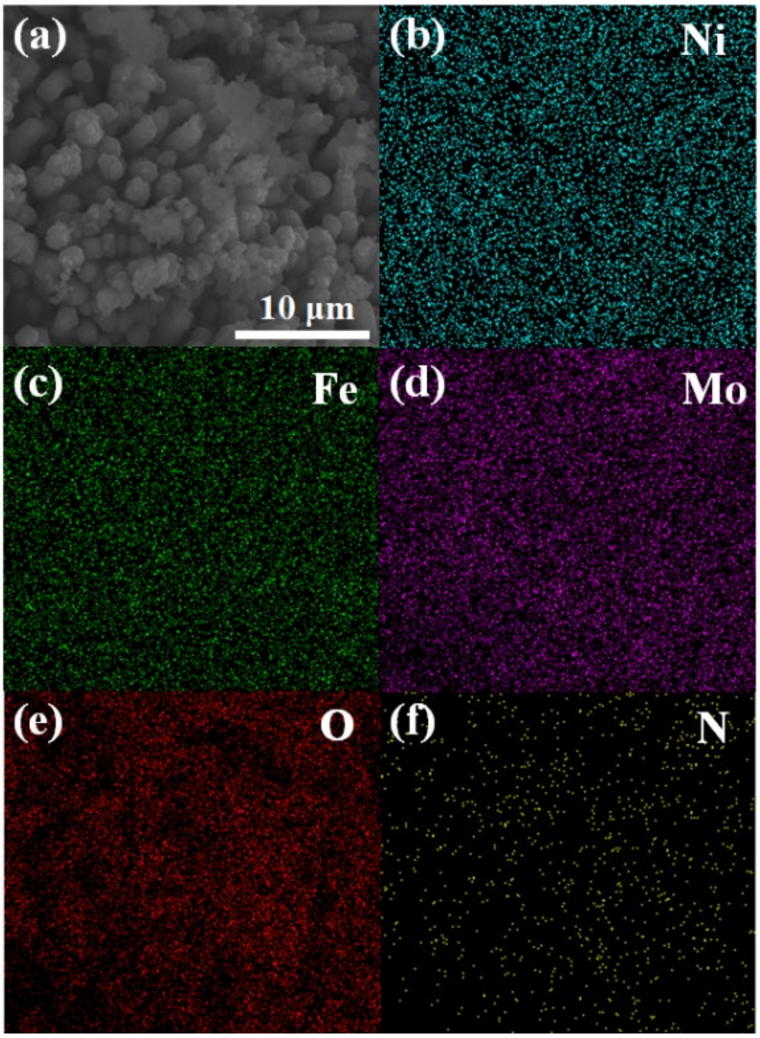

Supplementary Fig. 3. SEM image and element mapping of NiMoN/NiFe LDH.

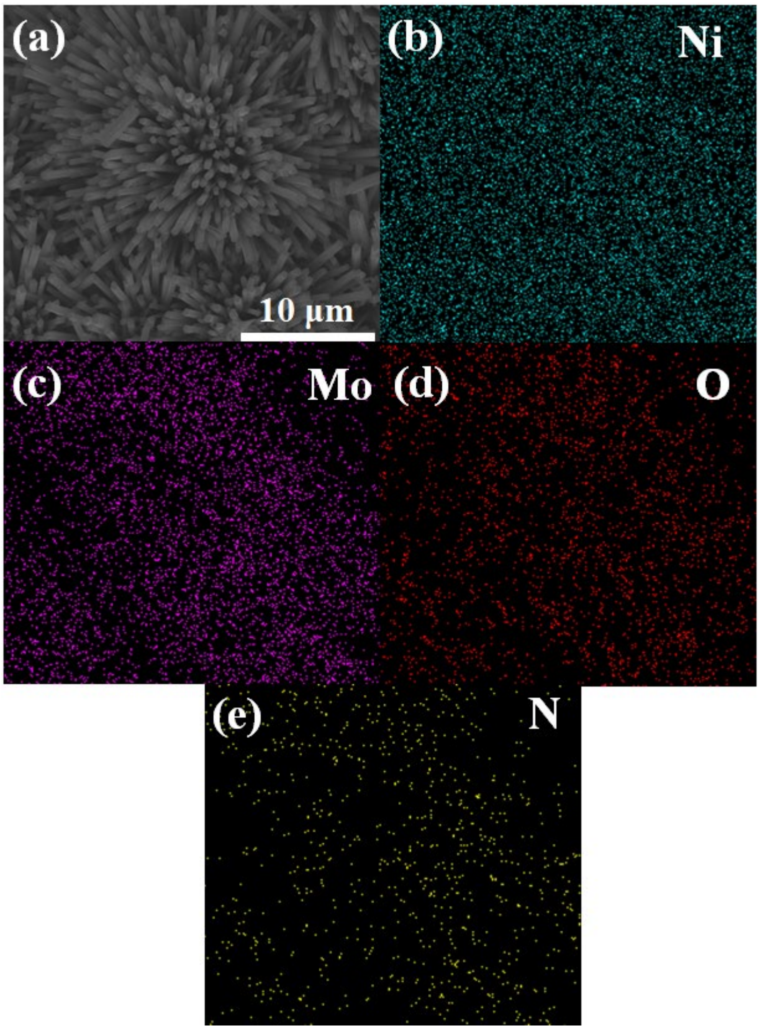

Supplementary Fig. 4. SEM image and element mapping of NiMoN.

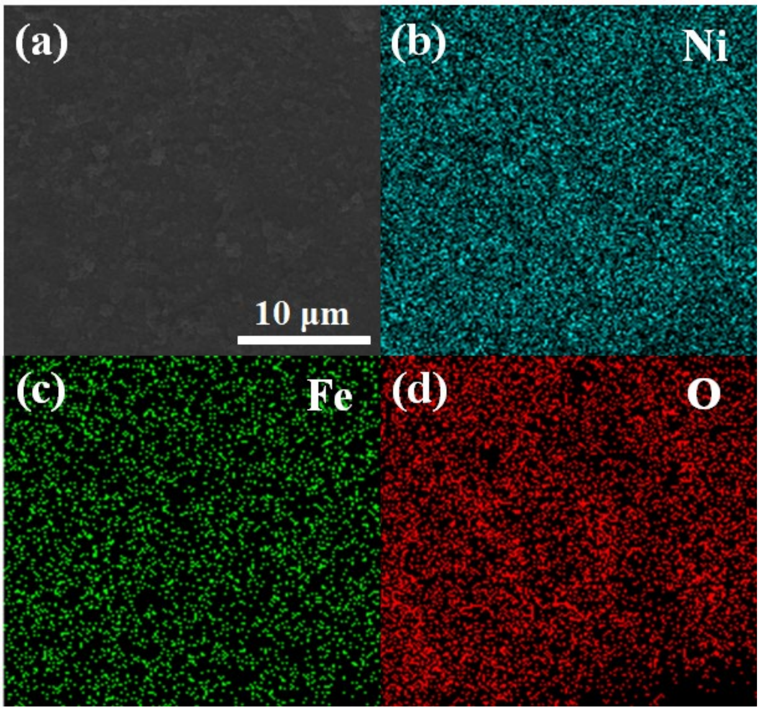

Supplementary Fig. 5. SEM image and element mapping of NiFe LDH.

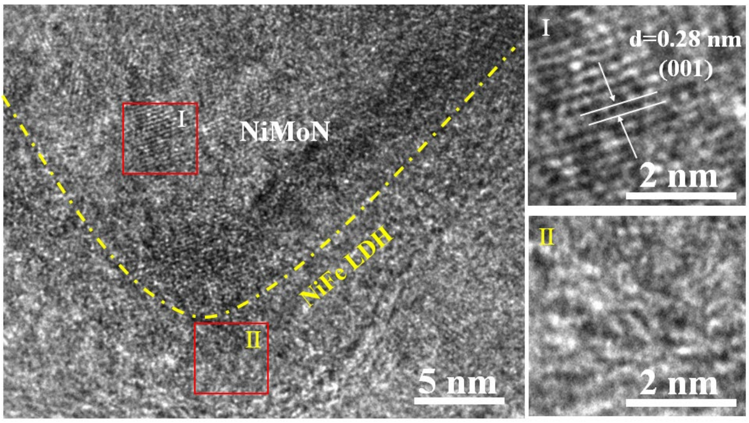

Supplementary Fig. 6. HR-TEM image of NiMoN/NiFe LDH.

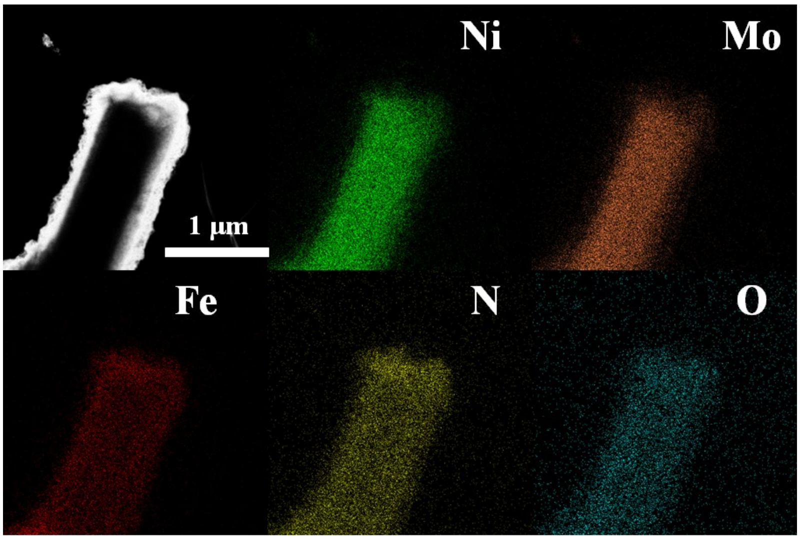

Supplementary Fig. 7. HAADF-STEM image of NiMoN/NiFe LDH and corresponding element mapping of Ni, Mo, Fe, N and O.

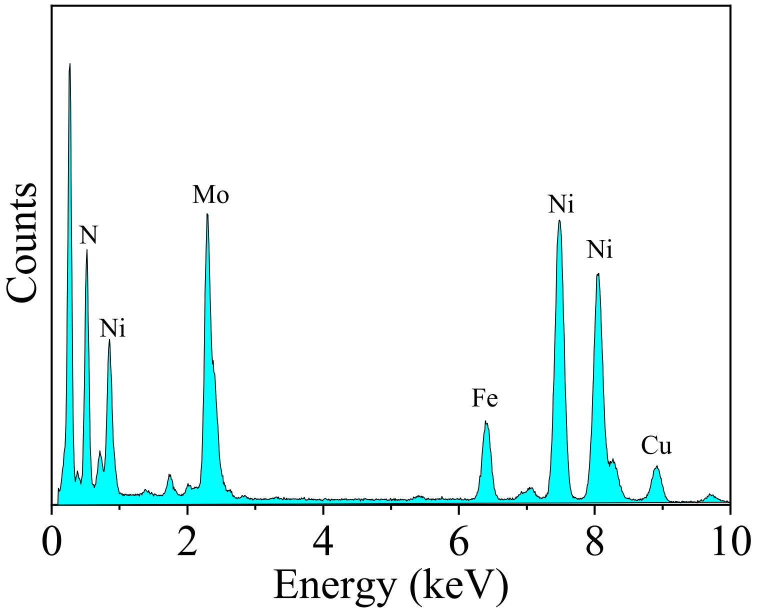

Supplementary Fig. 8. EDS spectrum of NiMoN/NiFe LDH.

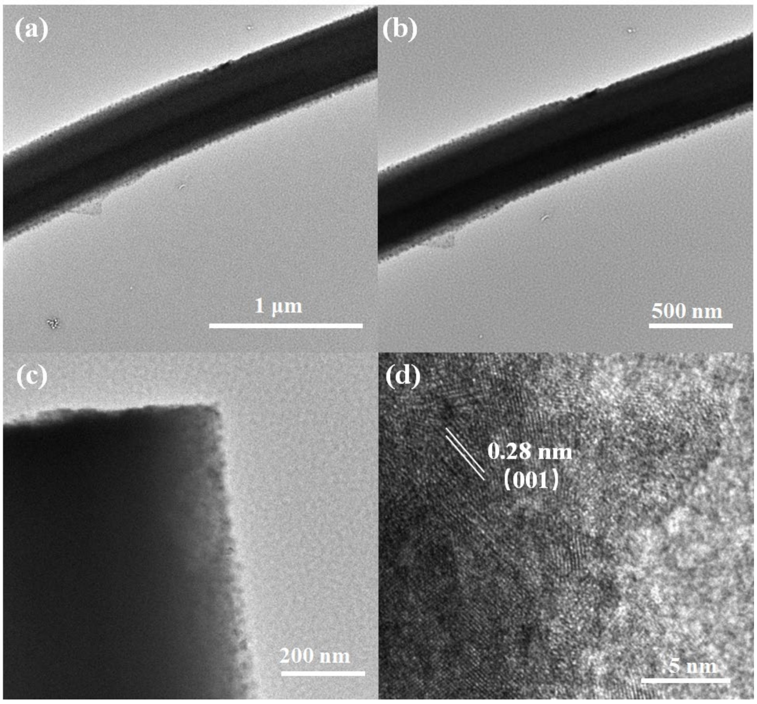

Supplementary Fig. 9. (a-c) TEM and (d) HR-TEM images of NiMoN nanorods.

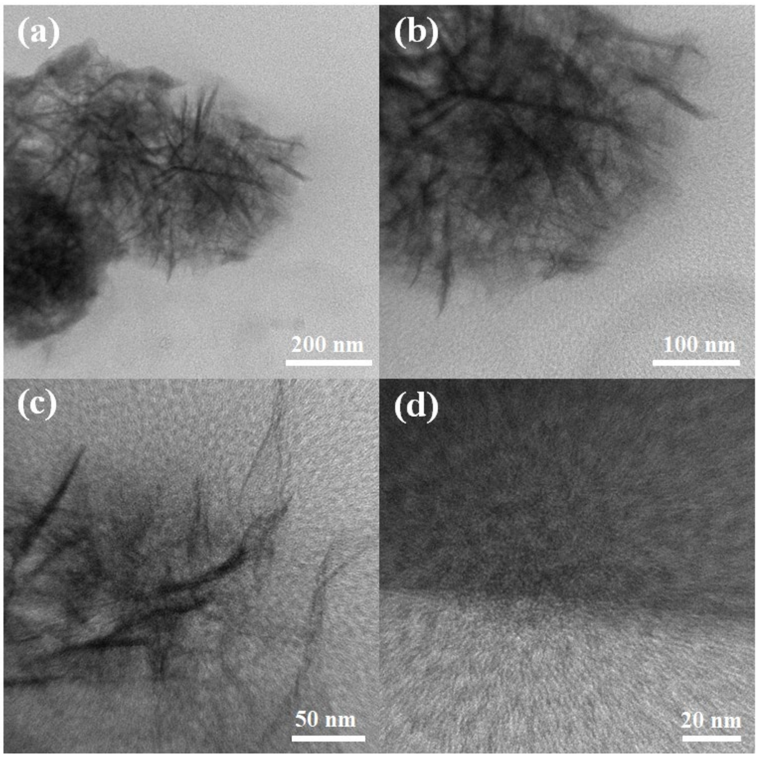

Supplementary Fig. 10. TEM images of NiFe LDH nanosheets.

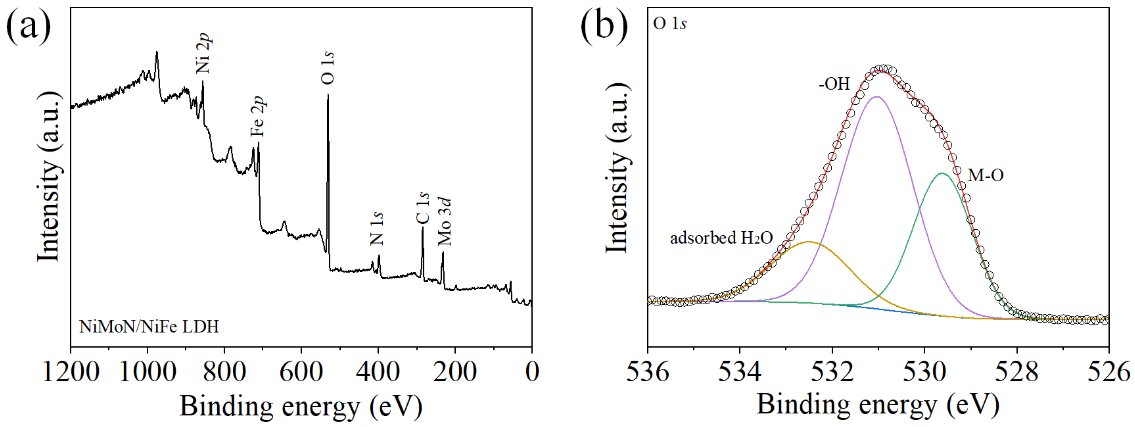

Supplementary Fig. 11. (a) XPS survey spectrum of NiMoN/NiFe LDH. (b) The high-resolution spectra of O 1*s*.

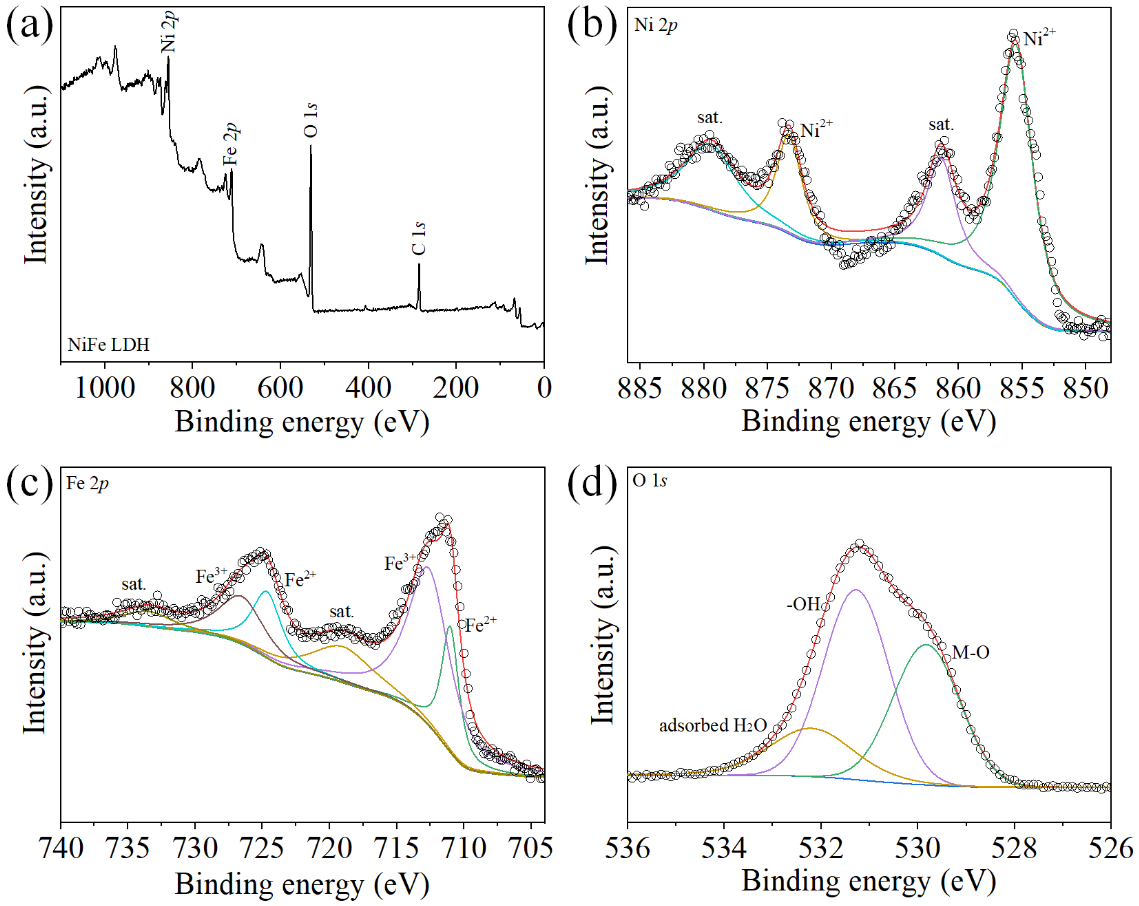

Supplementary Fig. 12. (a) XPS survey spectrum of NiFe LDH. The high-resolution spectra of (b) Ni 2*p*, (c) Fe 2*p* and (d) O 1*s*.

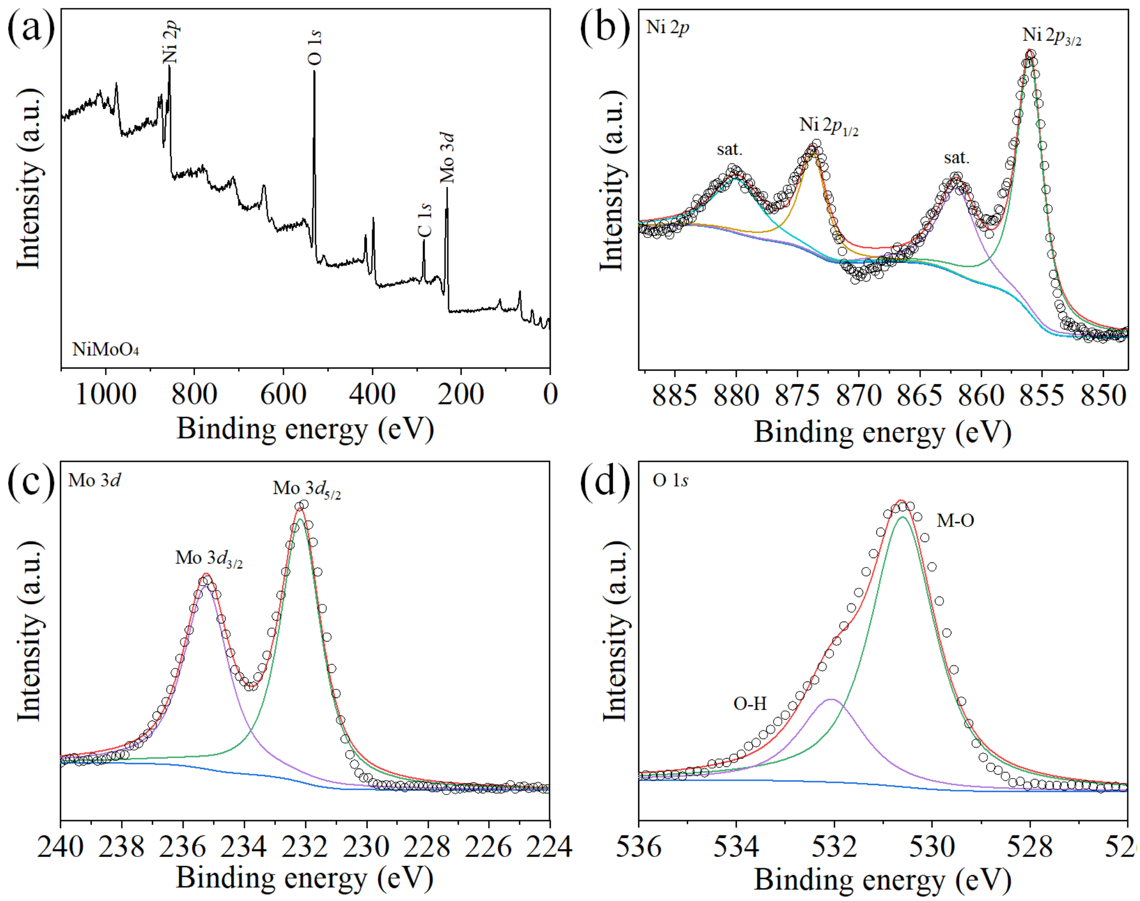

Supplementary Fig. 13. (a) XPS survey spectrum of NiMoO·H_2O. The high-resolution spectra of (b) Ni 2*p*, (c) Mo 3*d* and (d) O 1*s*.

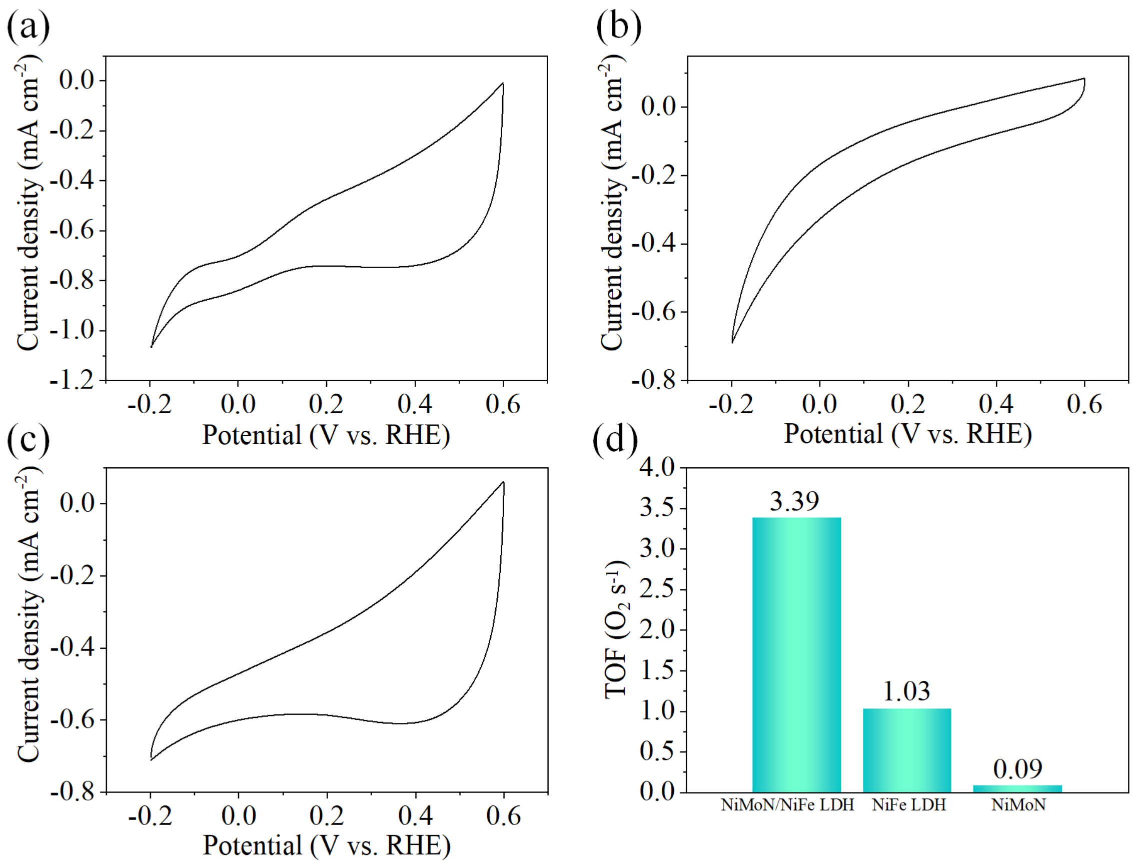

Supplementary Fig. 14. CV curve for (a) NiMoN/NiFe LDH, (b) NiFe LDH and (c) NiMoN recorded between -0.2 and 0.6 V vs. RHE in PBS (pH=7) at a scan rate of 50 mV s^{-1}. (d) Comparison of TOF value of NiMoN/NiFe LDH, NiFe LDH and NiMoN.

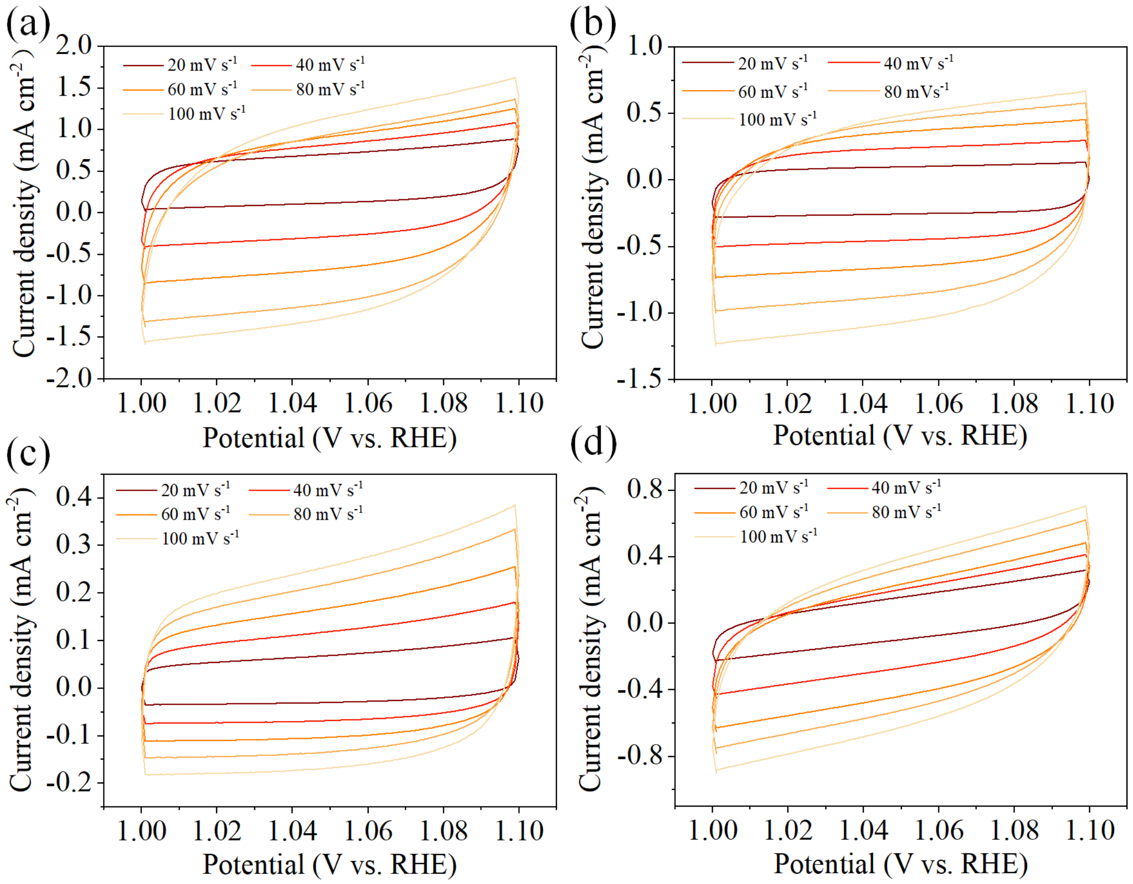

Supplementary Fig. 15. Cyclic voltammogram of (a) NiMoN/NiFe LDH, (b) NiFe LDH, (c) NiMoN and (d) $NiMoO_4{\cdot}H_2O$ at various scan rates (20, 40, 60, 80, 100 mV s^{-1}) in the non-Faradic region (1.0-1.1 V vs. RHE).

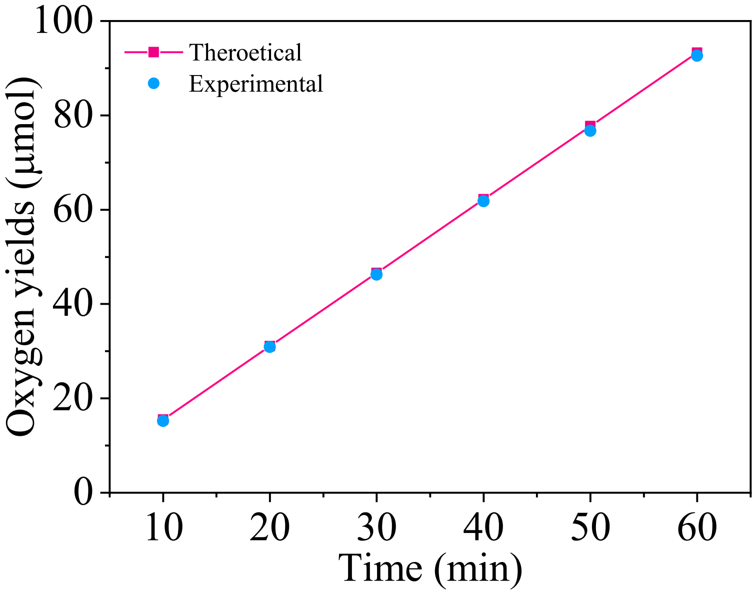

Supplementary Fig. 16. Theoretical and measured product yield for NiMoN/NiFe LDH at the current density of 10 mA cm^{-2}.

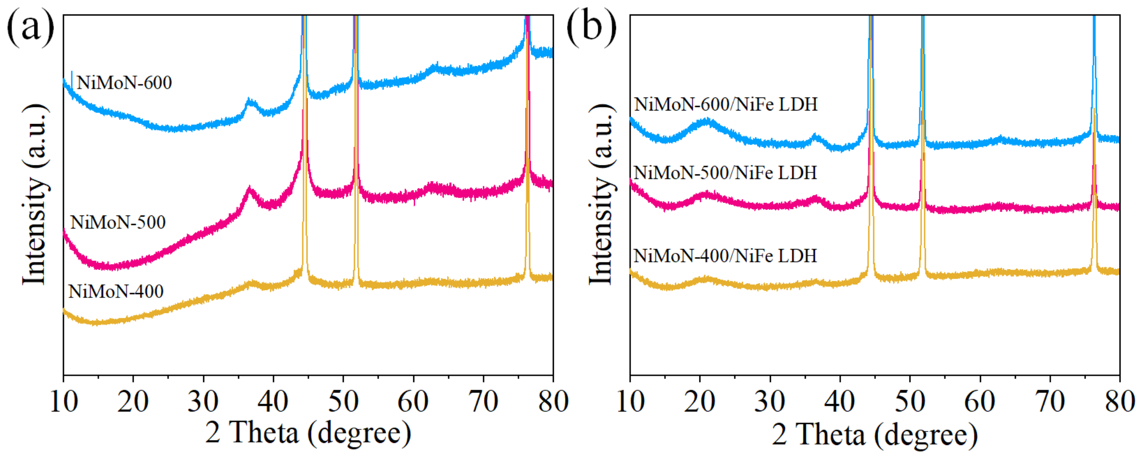

Supplementary Fig. 17. (a) The XRD patterns of NiMoN at different temperatures in NH_3 atomsphere. (b) The XRD patterns of NiMoN-T/NiFe LDH (T represents the nitridation temperature).

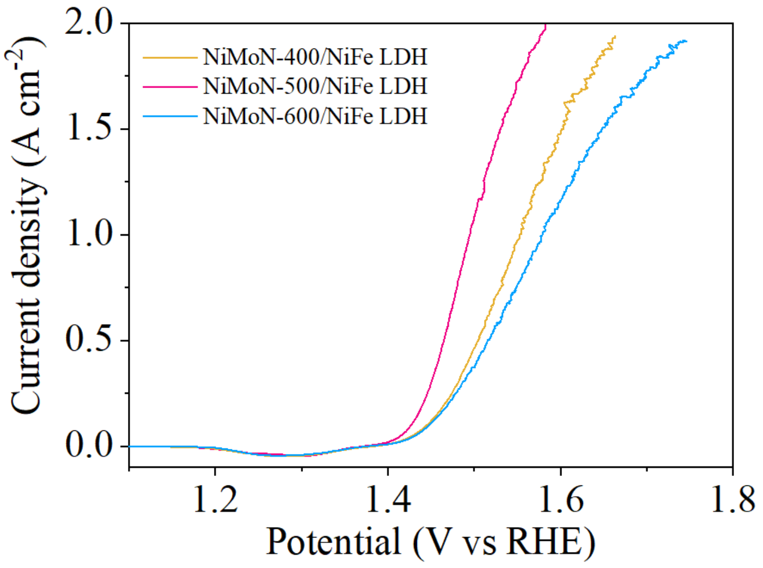

Supplementary Fig. 18. Polarization curves for OER of NiMoN-T/NiFe LDH (T represents the nitridation temperature).

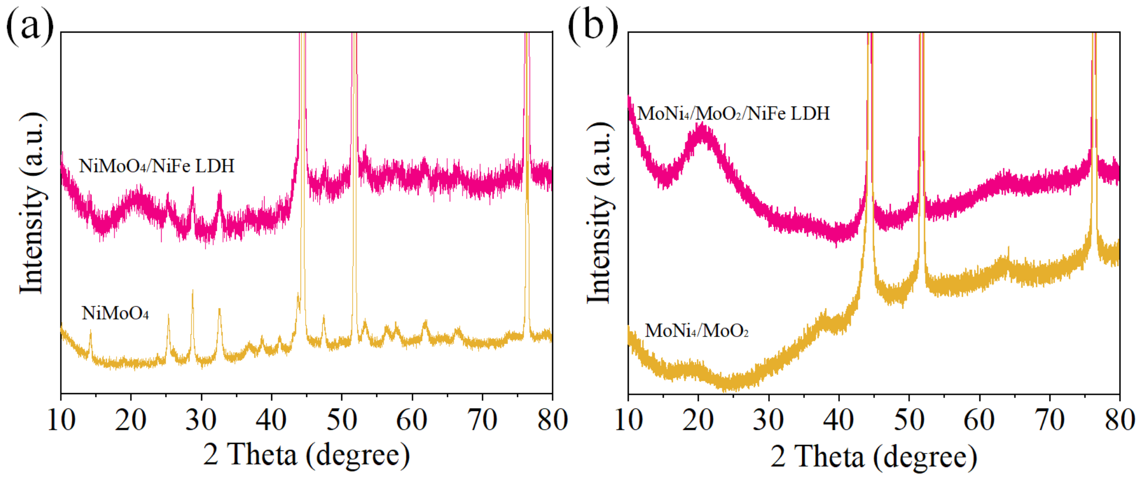

Supplementary Fig. 19. (a) The XRD patterns of $NiMoO_4$ and $NiMoO_4$/NiFe LDH. (b) The XRD patterns of $MoNi_4/MoO_2$ and $MoNi_4/MoO_2$/NiFe LDH.

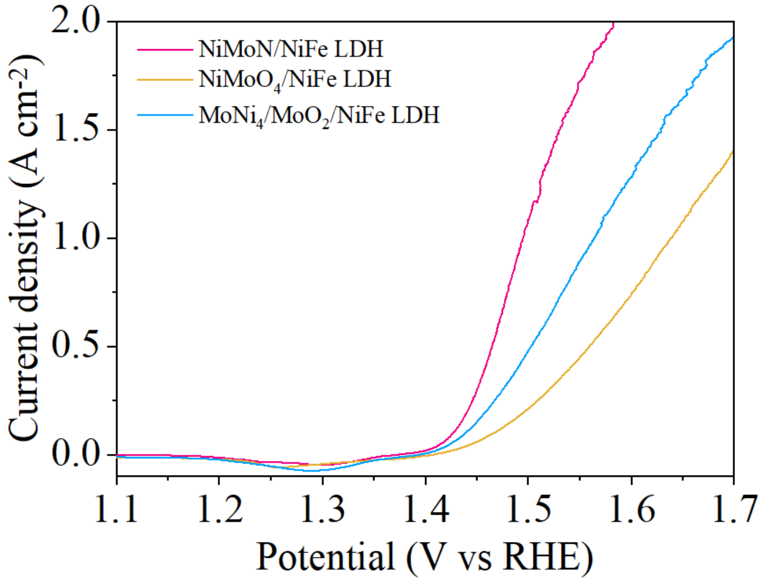

Supplementary Fig. 20. Polarization curves for OER of NiMoN/NiFe LDH, $MoNi_4/MoO_2$/NiFe LDH and $NiMoO_4$/NiFe LDH.

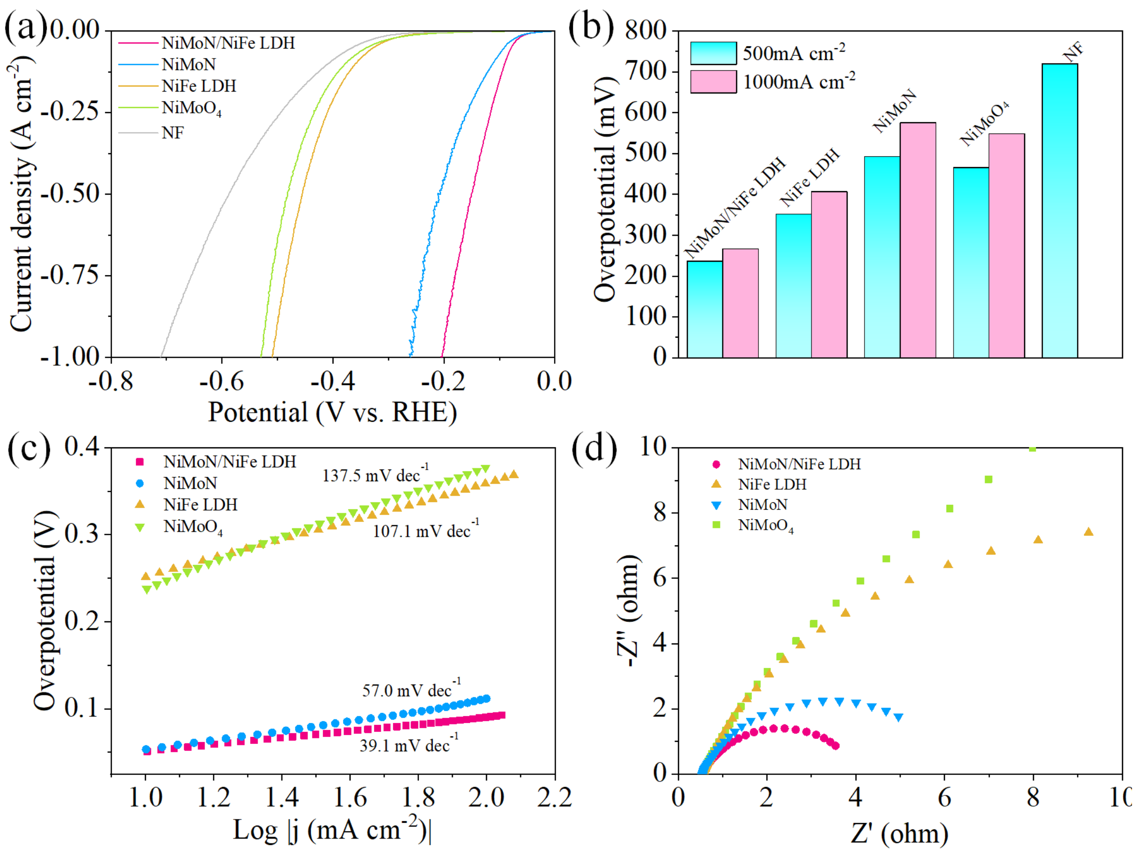

Supplementary Fig. 21. (a) The HER polarization curves of NiMoN/NiFe LDH, NiFe LDH, NiMoN, $NiMoO_4$ and Ni foam in 1 M KOH. (b) The overpotential of as-obtained samples at different current densities. (c)The Tafel slope of NiMoN/NiFe LDH, NiMoN, NiFe LDH and $NiMoO_4$. (d) Electrochemical impedance spectroscopy.

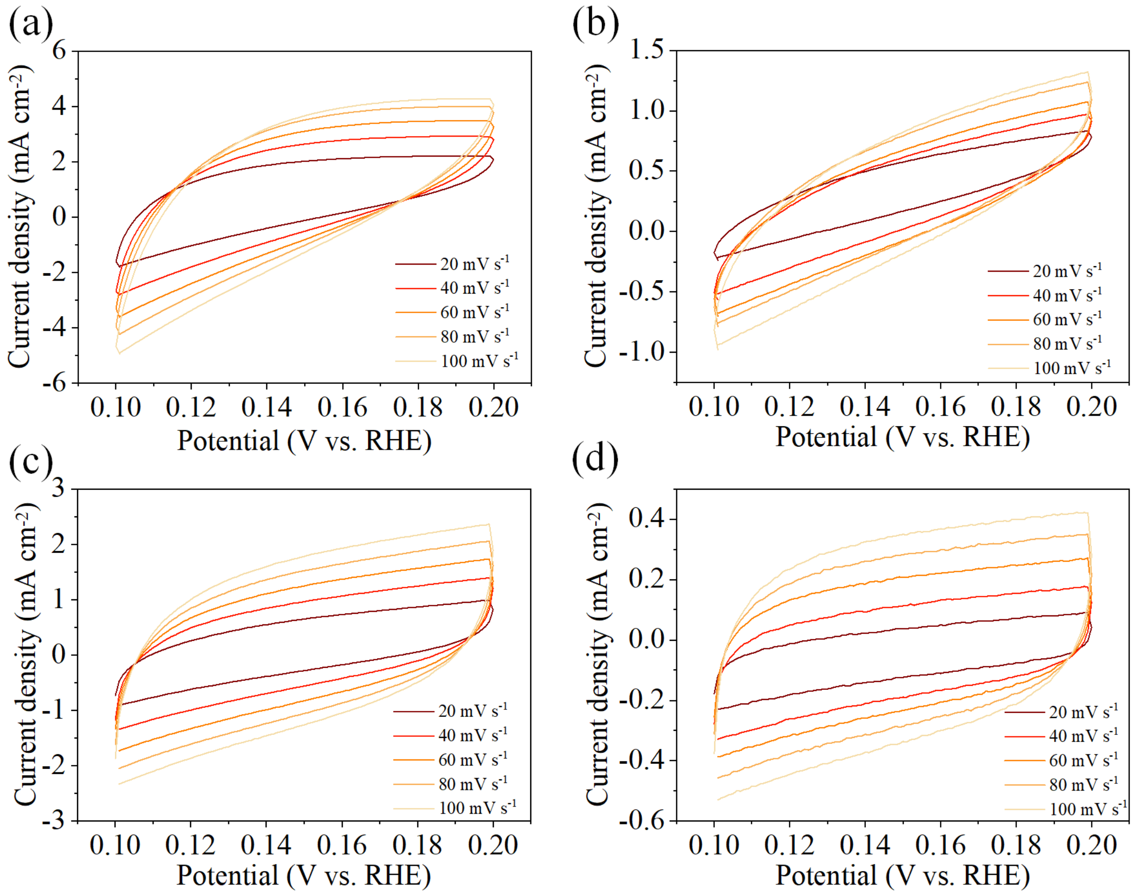

Supplementary Fig. 22. Cyclic voltammogram of (a) NiMoN/NiFe LDH, (b) NiFe LDH, (c) NiMoN and (d) $NiMoO_4 \cdot H_2O$ at various scan rates (20, 40, 60, 80, 100 mV s^{-1}) in the non-Faradic region (0.1-0.2 V vs. RHE).

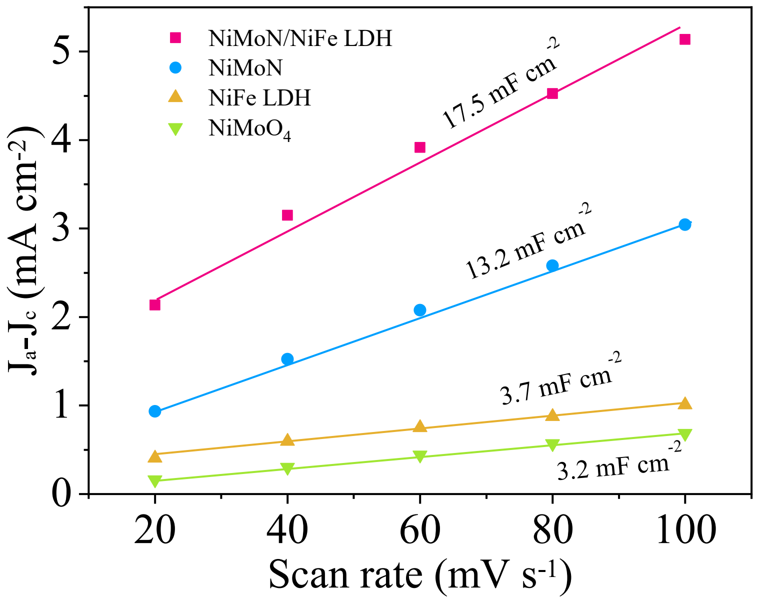

Supplementary Fig. 23. Double-layer capacitance (C_{dl}) of as-obtained electrocatalysts.

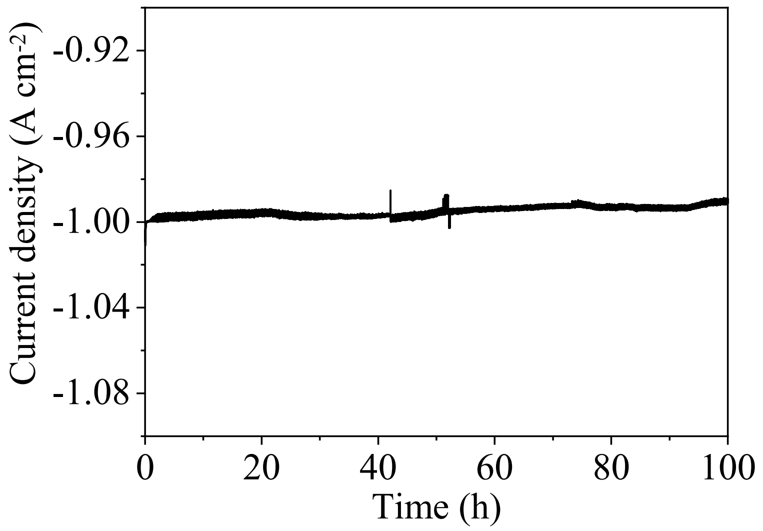

Supplementary Fig. 24. The chronoamperometry curve of NiMoN/NiFe LDH for a continuous 100 h operation at a constant potential.

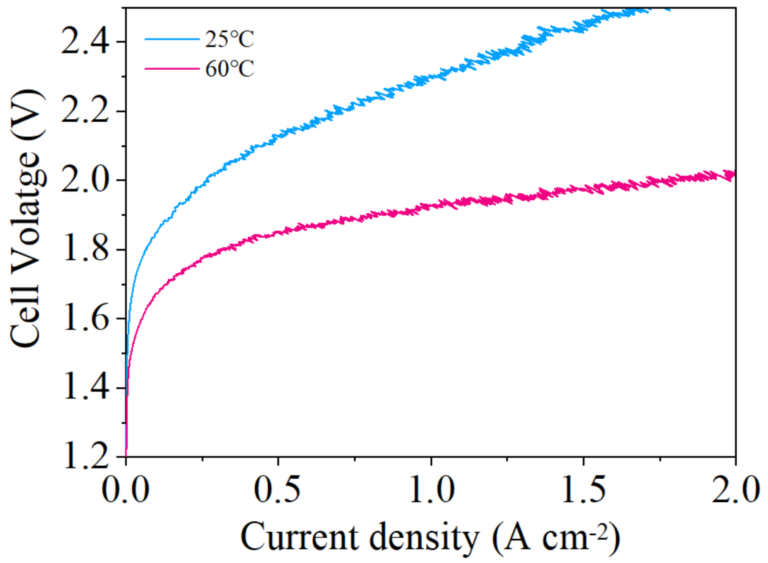

Supplementary Fig. 25. Electrocatalytic water splitting performance of NiMoN/NiFe LDH in MEA setup at different temperatures.

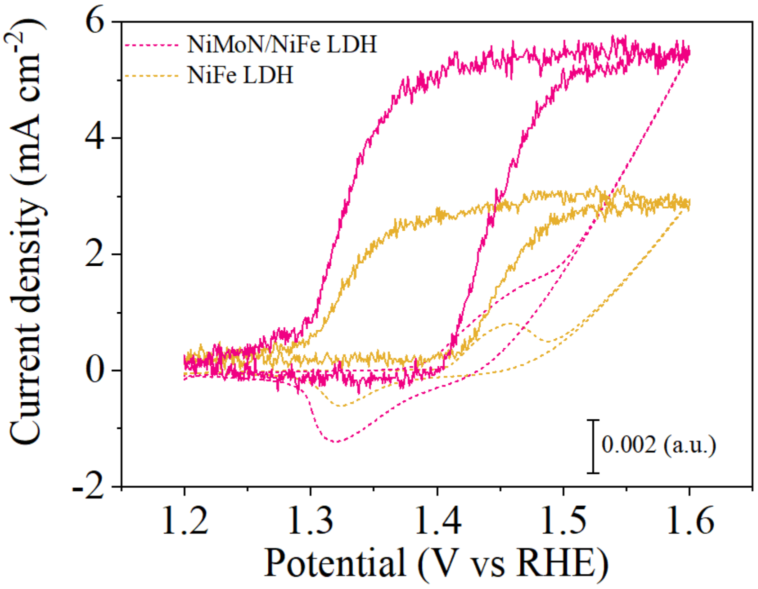

Supplementary Fig. 26. In-situ UV-Vis absorption spectra of NiMoN/NiFe LDH and NiFe LDH during potential cycling at the range of 1.2 to 1.6 V vs. RHE and corresponding CV curves.

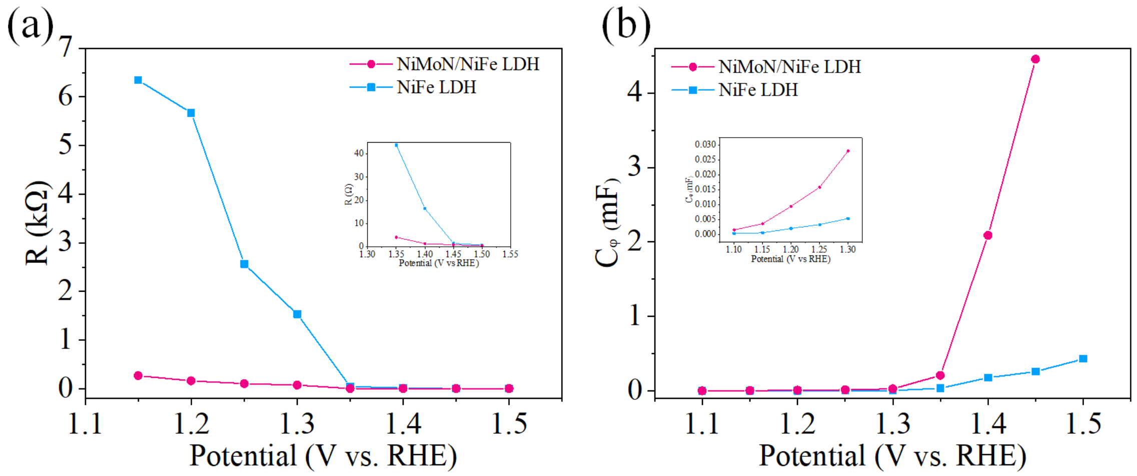

Supplementary Fig. 27. a) Plot of the resistance to the applied potential of NiMoN/NiFe LDH and NiFe LDH. b) Plot of CPE_{OH} versus applied potential of NiMoN/NiFe LDH and NiFe LDH.

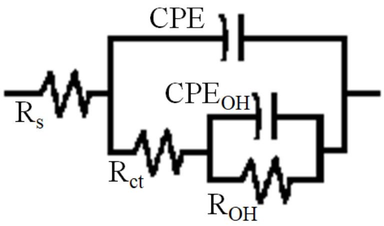

Supplementary Fig. 28. The equivalent circuit used for modeling the electrochemical impedance spectra. R_s represents solution resistance. Rct and R_{OH} are related with the interface reaction charge transfer resistances and resistances of the intermediate accumulation. CPE and CPE_{OH} are represented the double layer capacitance and the relaxation of the charge associated with adsorbed intermediate.

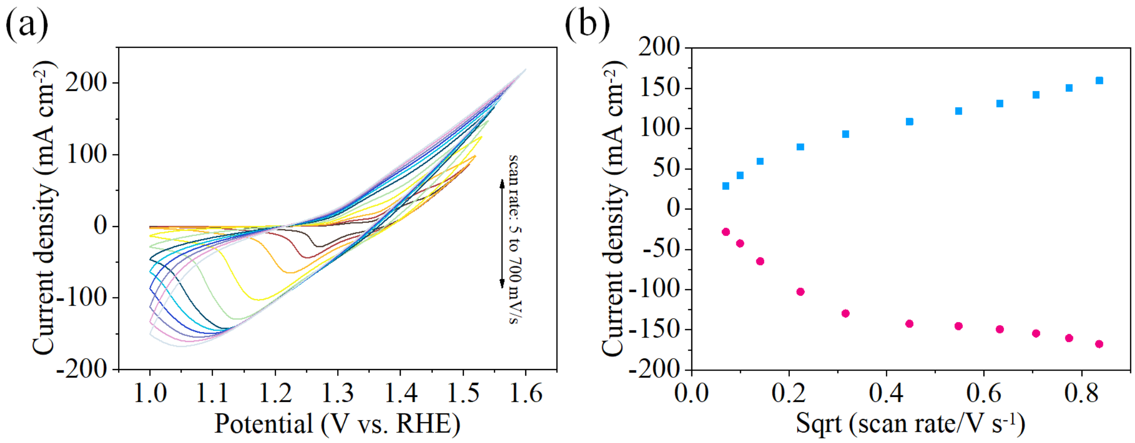

Supplementary Fig. 29. Analysis of NiMoN/NiFe LDH in Laviron equation. a) CVs of NiMoN/NiFe LDH with scan rates from 5, 10, 20, 50, 100, 200, 300, 400, 500, 600 to 700 mV s^{-1} in 1 M KOH. b) The plot of the redox peak currents densities versus the square root of scan rates.

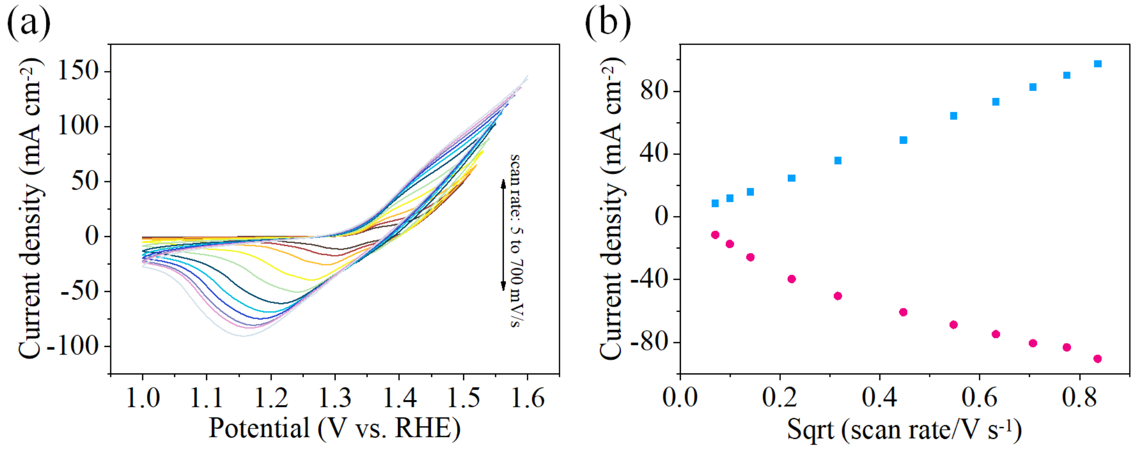

Supplementary Fig. 30. Analysis of NiFe LDH in Laviron equation. a) CVs of NiFe LDH with scan rates from 5, 10, 20, 50, 100, 200, 300, 400, 500, 600 to 700 mV s^{-1} in 1 M KOH. b) The plot of the redox peak currents densities versus the square root of scan rates.

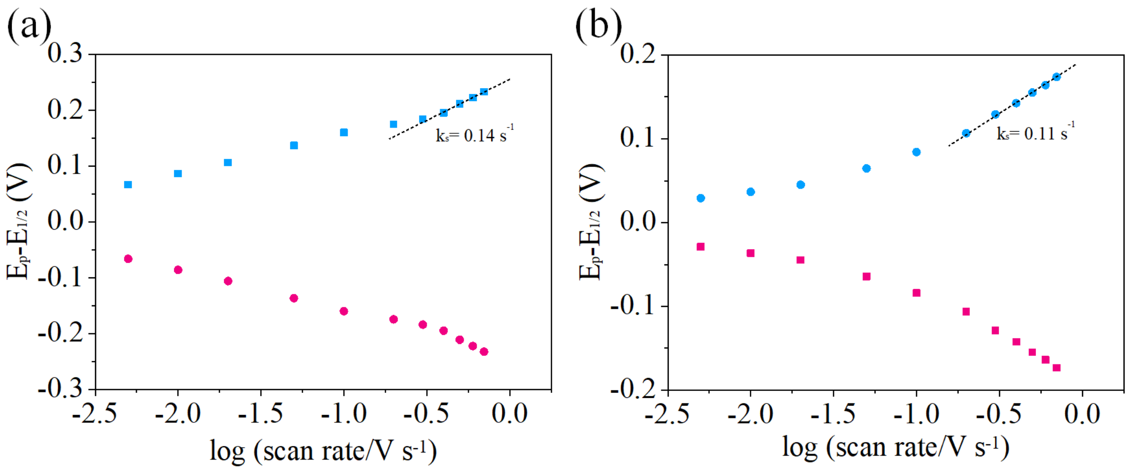

Supplementary Fig. 31. The plot of the redox peak potentials versus the logarithm of scan rate of a) NiMoN/NiFe LDH and b) NiFe LDH.

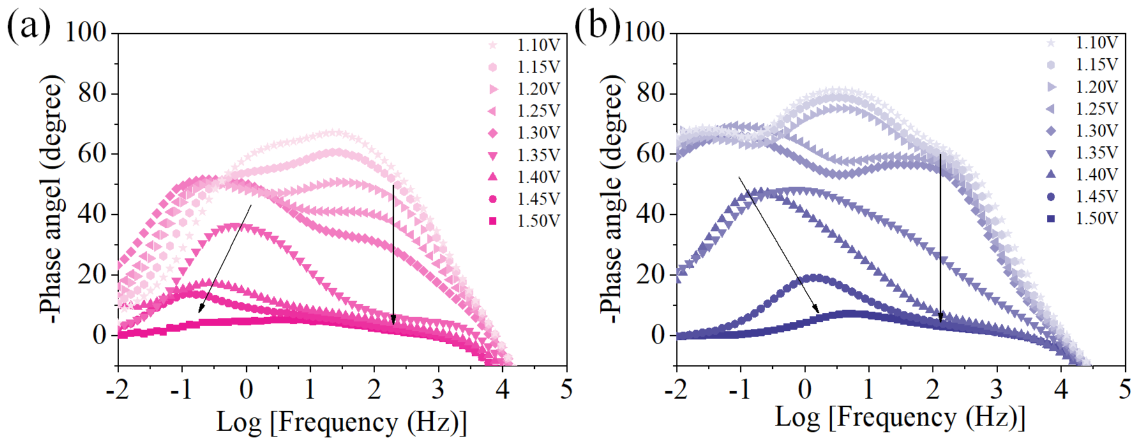

Supplementary Fig. 32. Bode phase plots of (a) NiMoN/NiFe LDH and (b) NiFe LDH at different applied potentials.

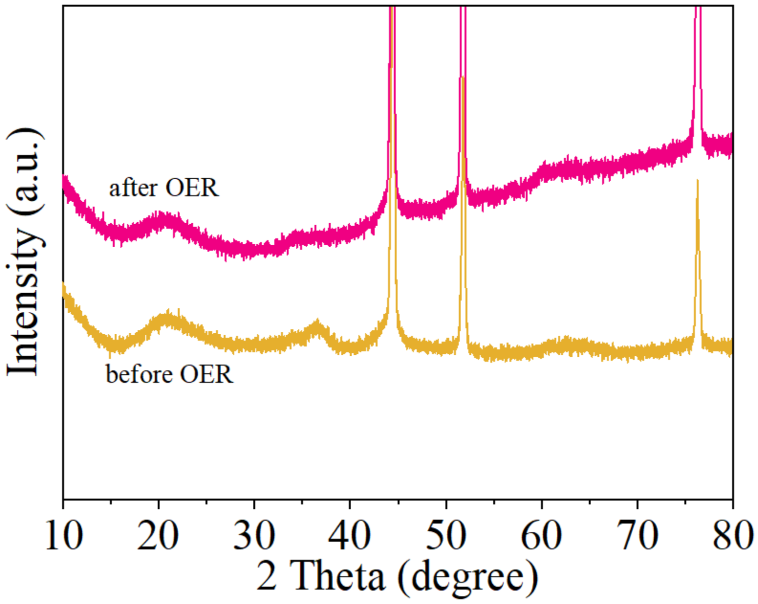

Supplementary Fig. 33. XRD patterns of NiMoN/NiFe LDH before and after OER.

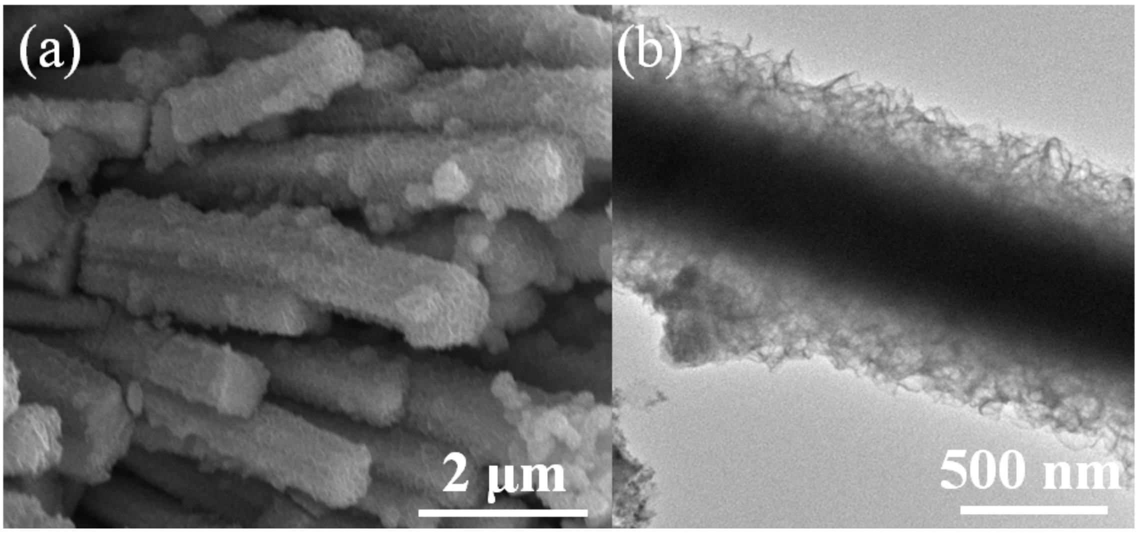

Supplementary Fig. 34. (a) SEM and (b) TEM image of NiMoN/NiFe LDH after OER.

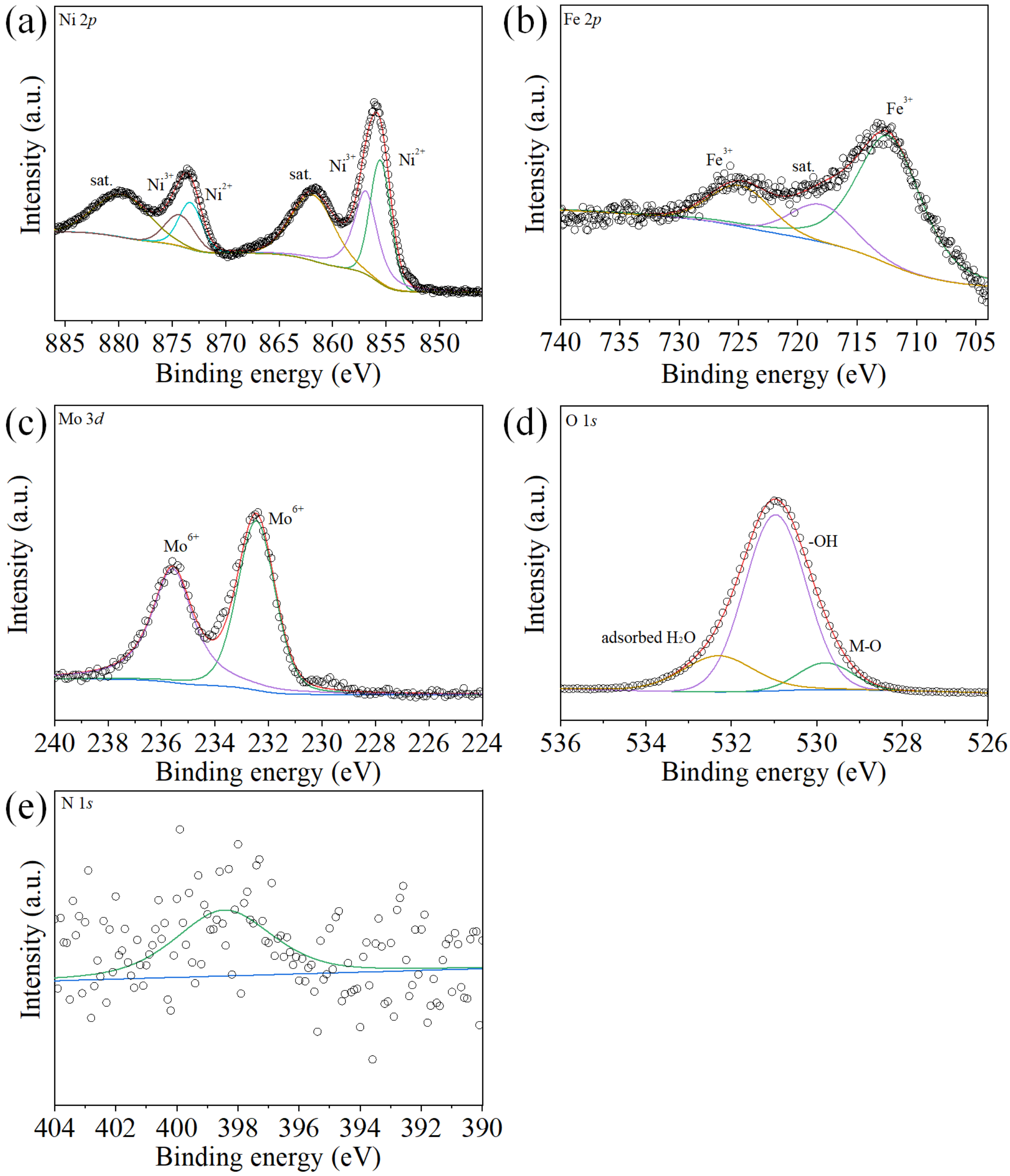

Supplementary Fig. 35. The high-resolution XPS spectra of (a) Ni 2*p*, (b) Fe 2*p*, (c) Mo 3*d*, (d) O 1*s* and (f) N 1*s.*

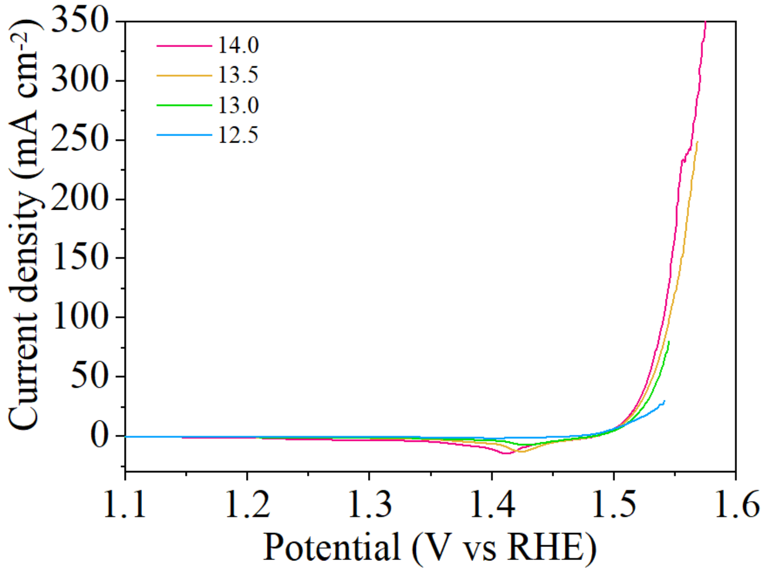

Supplementary Fig. 36. Linear sweep voltammetry curves of NiFe LDH in alkaline electrolytes with different pH.

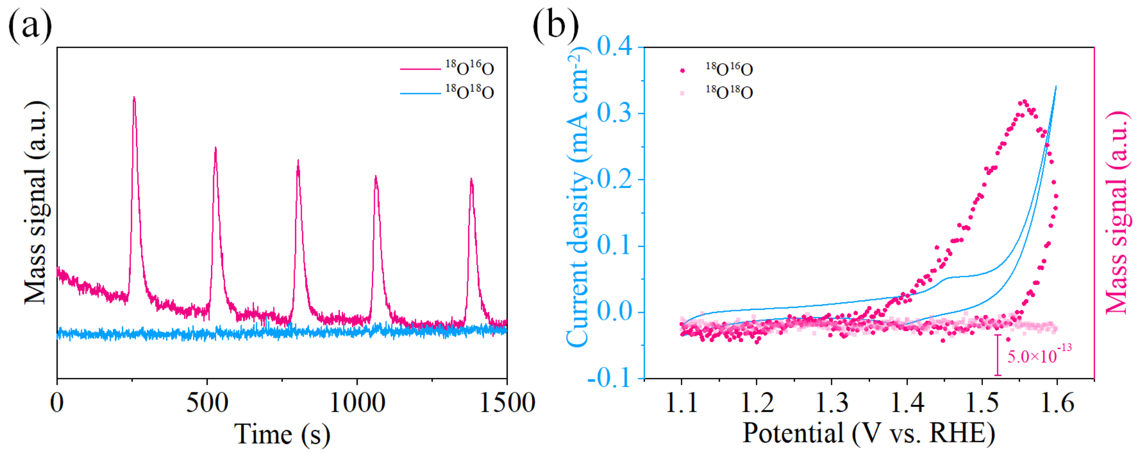

Supplementary Fig. 37. (a) Variation of DEMS signals of $^{34}O_2$ and $^{36}O_2$ for NiFe LDH versus time. (b) The DEMS signals of $^{34}O_2$ and $^{36}O_2$ and the CV curves for NiFe LDH versus applied potential.

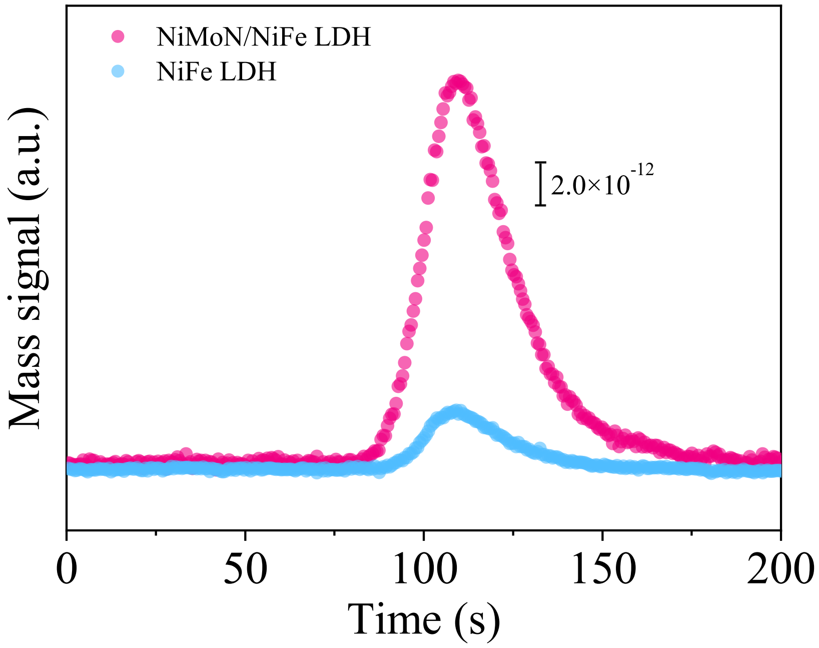

Supplementary Fig. 38. DEMS signals of $^{34}O_2$ versus time for NiMoN/NiFe LDH and NiFe LDH.

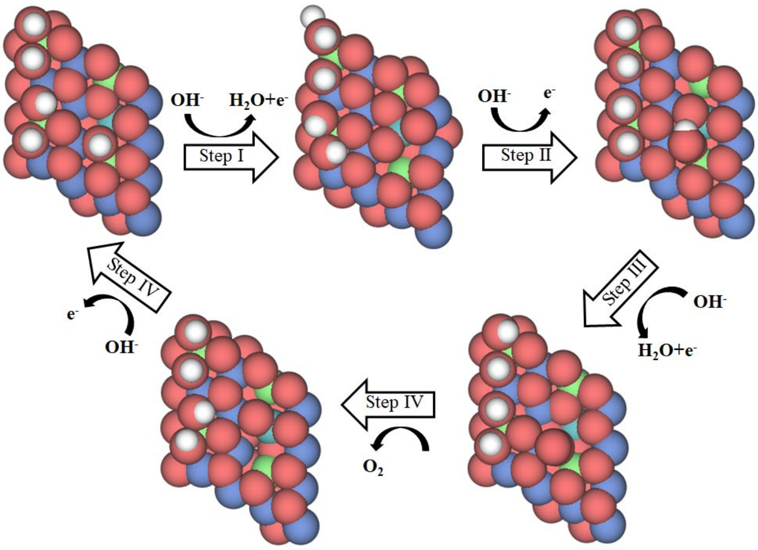

Supplementary Fig. 39. Schematic illustrations of LOM pathway.

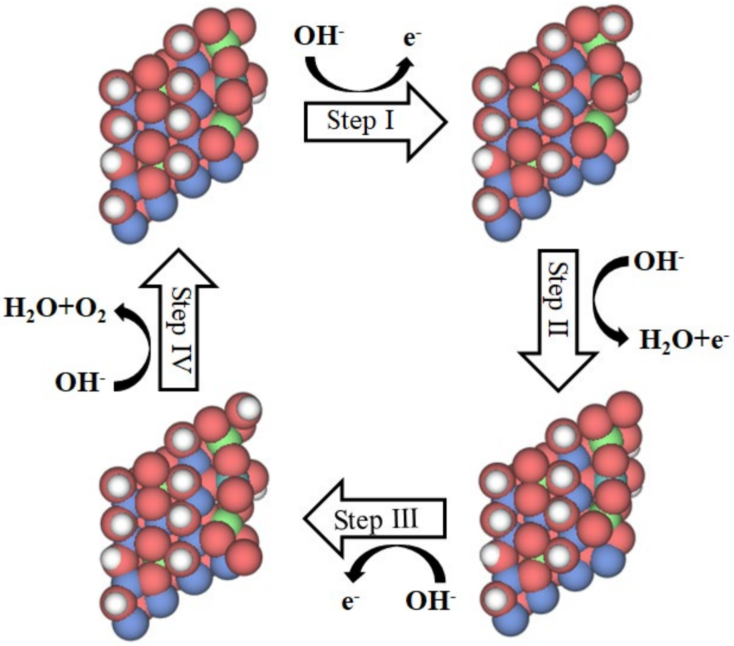

Supplementary Fig. 40. Schematic illustrations of AEM pathway.

Supplementary note 1

Alkaline anion exchange membrane water electrolysis (AEMWE) test:

NiMoN/NiFe LDH electrocatalysts was integrated into a MEA to assemble AEMWE device. PiperION-A40 (Versogen) was used as the anion exchange membrane and immersed in 1M KOH solution for 12h before used to exchange HCO_3^- into OH^-. NiMoN/NiFe LDH catalyst grown on Ni foam used both as anode and cathode. The gas diffusion layer (GDL) was prepared by pressing Ni foam. Electrically insulating gaskets were also placed to prevent the liquid and gas from escaping through any space between flow fields. Appropriate fastening pressure of the AEMWE cell was applied on four bolts. The performance of an AEMWE with an active area of 1 cm^2 was evaluated through LSV curves. The flowing 1 M KOH solution was used as electrolyte and the temperature of electrolyte can be controlled at 20 to 60 ℃ by a constant temperature heating chamber through a peristaltic pump. The potential range is from 1.2 to 2.5 V at a scan rate of 5 mV s^{-1}.

Supplementary note 2

First-principle calculation: DFT calculations were carried out using the Vienna Ab-initio Simulation Package (VASP)[1]. The Perdew-Burke-Ernzerhof (PBE) functional within the generalized gradient approximation (GGA) was used to describe the exchange-correlation interactions. The electron-ion interactions were described by projector augmented wave (PAW) potentials[2]. A kinetics energy cut-off of 450 eV was used. The force and energy convergence criteria were set as 0.02 eV $Å^{-1}$ and 10^{-4} eV. The Brillouin zone was sampled with a 3×3×1 Monkhorst-Pack grid. The DFT-D3 method was used to evaluate the van der Waals (vdW) correction[3]. The Hubbard-U terms for Ni and Fe were considered, with the effective U value of 4.0 and 4.3 eV for Ni and Fe, respectively. The water solvation effect was also considered by using VASPsol[4]. The COHP of considered atomic pairs was calculated by the Lobster code[5]. Good accuracy of all COHP calculations was obtained with the charge spilling lower than 1%.

In an alkaline environment, The Gibbs free energy of the adsorbed substance can be calculated as:

$$\Delta G = E_{\mathrm{ads}} + \Delta E_{\mathrm{ZPE}} - T\Delta S - \Delta G(\mathrm{pH}) + eU \qquad (1)$$

where E_{ads} is the adsorption energy of intermediate, ΔE_{ZPE} is the zero point energy difference between the adsorption state and gas state, T is the temperature, ΔS is the entropy various between the adsorption and gas phase. For adsorbates, E_{ZPE} and S are obtained from vibrational frequencies calculations with harmonic approximation and contributions from the slabs are neglected, whereas for molecules these values are taken from the NIST database. $\Delta G(\mathrm{pH}) = \mathrm{k}T \ln 10 * \mathrm{pH}$ is the contribution of the pH in solutions different from 0. where k is the Boltzmann constant. U is the potential measured against normal hydrogen electrode (NHE) at standard condition (P = 1 bar, pH = 0).

In alkaline conditions，the Gibbs free energy changes for the water oxidation steps using AEM mechanism was calculated using the following Equations (2-5) of four steps[6]:

$$*OH + OH^- \rightarrow *O + H_2O + e^- \qquad (2)$$

$$*O + OH^- \rightarrow *OOH + e^- \qquad (3)$$

$$*OOH + OH^- \rightarrow *OO + H_2O + e^- \qquad (4)$$

$$*OO + OH^- \rightarrow *OH + O_2 + e^- \qquad (5)$$

where * denotes adsorption active site on the substrate.

The LOM pathway includes five steps (6-10), which are[7]:

$$*O_lH + OH^- \rightarrow *O_l + H_2O + e^- \quad (6)$$

$$*O_l + OH^- \rightarrow *O_lOH + e^- \quad (7)$$

$$*O_lOH + OH^- \rightarrow *O_lO + H_2O + e^- \quad (8)$$

$$*O_lO \rightarrow * + O_2 \quad (9)$$

$$* + OH^- \rightarrow *O_lOH + e^- \quad (10)$$

where “*” represents the vacancy sites. O_l denotes the lattice oxygen atoms.

Supplementary note 3

The DEMS experiments: The DEMS experiments were performed using QAS 100 mass spectrometer (Linglu Instruments, Shanghai). The DEMS experiments were carried out with a custom-made electrochemical cell, in which Pt wire and Ag/AgCl electrode used as counter electrode and the reference electrode, respectively. The working electrode was prepared by drop-casting the catalysts ink on a gold disk electrode with 8 mm diameter and loading of 0.1 mg cm^{-2}. Firstly, the working electrodes were isotopically labeled for 10 min at 1.52 V vs. RHE in 0.1 M KOH with $H_2{}^{18}O$ (97% ^{18}O, Wuhan Isotope Technology Co., Ltd.). Afterward, the ^{18}O-labeled electrodes were rinsed with $H_2{}^{16}O$ and measured at the potential range of 1.1-1.6 V vs. RHE in 0.1 M KOH with $H_2{}^{16}O$ using CV with a scan rate of 5 mV s^{-1}. Meanwhile, the generated gas products were constantly monitored by mass spectrometry.

Supplementary Table 1. Comparison of OER activity data for various catalysts.

Catalysts	Current density (mA cm^{-2})	Overpotential (mV)	References
Se-doped FeOOH	500	348	J. Am. Chem. Soc., 141, 7005 (2019)
FeP/Ni_2P	500	280	Nat. Commun., 9, 2551 (2018)
$NiMoO_x$/ NiMoS	500	278	Nat. Commun., 11, 5462 (2020)
Cu@NiFe LDH	500	315	Energy Environ. Sci., 10, 1820 (2017)
$Ni_{0.8}Fe_{0.2}$–AHNA	500	248	Energy Environ. Sci., 13, 86 (2020)
$Ni_{83}Fe_{17}$-ONCAs	500	255	Adv. Mater., 33, 2007377 (2021)
Co_9S_8@Fe_3O_4	500	350	ACS Catal., 12, 4318 (2022)
Fe-CoP/NF	500	295	Adv. Sci., 5, 1800949 (2018)
Co_1Mn_1CH	1000	462	J. Am. Chem. Soc., 139, 8320 (2017)
Fe_{MOFs}-SO_3	1000	330	Adv. Energy Mater., 10, 2000184 (2020)
NiFe LDH/NiS	1000	325	Adv. Energy Mater., 11, 2102353 (2021)
$FeNiCoCrMnS_2$	1000	308	Adv. Funct. Mater., 31, 2106229 (2021)
**NiMoN/NiFe LDH**	**500**	**236**	**This work**
**NiMoN/NiFe LDH**	**1000**	**266**	**This work**

Supplementary Table 2. Comparison of overall water splitting data for various catalysts.

Catalysts	Current density (mA $cm^{-2)}$	Cell Voltage (V)	References
CoMoSx/NF	500	1.89	Angew. Chem. Int. Ed., 59, 1659 (2020)
FeP/Ni_2P	500	1.72	Nat. Commun., 9, 2551 (2018)
$NiMoO_x$/ NiMoS	500	1.75	Nat. Commun., 11, 5462 (2020)
$Ni_{0.8}Fe_{0.2}$–AHNA// Ni nanowire array	500	1.70	Energy Environ. Sci., 13, 86 (2020)
hier-NiFe@sCNTs	500	1.71	Small, 16, 2002511 (2020)
Co_4N-CeO_2	500	1.99	Adv. Funct. Mater., 30, 1910596 (2020)
NiFe-LDH/MXene/ NF	500	1.75	Nano Energy, 63, 103880 (2019)
Co_4N-CeO_2	1000	2.28	Adv. Funct. Mater., 30, 1910596 (2020)
Ru, Ni-CoP	1000	1.833	Appl. Catal. B, 298, 120488 (2021)
MnO_x/NiFeP/NF	1000	1.828	Small, 18, 2105803 (2022)
Ru-CoO_x/NF	1000	2.2	Small, 17, 2102777 (2021)
**NiMoN/NiFe LDH**	**500**	**1.70**	**This work**
**NiMoN/NiFe LDH**	**1000**	**1.77**	**This work**

Supplementary Reference

1. Perdew, J. P., Burke, K. & Ernzerhof, M. Generalized gradient approximation made simple. *Phys. Rev. Lett.* **78**, 1396–1396 (1997).
2. Blochl, P. E. Projector augmented-wave method. *Phys. Rev. B* **50**, 17953 (1994).
3. Grimme, S., Ehrlich, S. & Goerigk, L. Effect of the damping function in dispersion corrected density functional theory. *J. Comput. Chem.* **32**, 1456-1465 (2011).
4. Mathew, K., Sundararaman, R., Letchworth-Weaver, K., Arias, T. A. & Hennig, R. G. Implicit solvation model for density-functional study of nanocrystal surfaces and reaction pathways. *J. Chem. Phys.* **140**, 084106 (2014).
5. Dronskowski, R. & Blochl, P. E. Crystal orbital hamilton populations (COHP): energy-resolved visualization of chemical bonding in solids based on density-functional calculations. *J. Phys. Chem.* **97**, 8617–8624 (1993).
6. Grimaud, A. et al. Activating lattice oxygen redox reactions in metal oxides to catalyse oxygen evolution. *Nat. Chem.* **9**, 457-465 (2017).
7. He, Z., Zhang, J., Gong, Z. et al. Activating lattice oxygen in NiFe-based (oxy)hydroxide for water electrolysis. *Nat. Commun.* **13**, 2191 (2022).
